# Shared control of a 16 semiconductor quantum dot crossbar array

**DOI:** 10.1038/s41565-023-01491-3

**Published:** 2023-08-28

**Authors:** Francesco Borsoi, Nico W. Hendrickx, Valentin John, Marcel Meyer, Sayr Motz, Floor van Riggelen, Amir Sammak, Sander L. de Snoo, Giordano Scappucci, Menno Veldhorst

**Affiliations:** 1 QuTech and Kavli Institute of Nanoscience, Delft University of Technology, Delft, The Netherlands; 2 QuTech and Netherlands Organisation for Applied Scientific Research (TNO), Delft, The Netherlands

**Keywords:** Qubits, Electronic devices

## Abstract

The efficient control of a large number of qubits is one of the most challenging aspects for practical quantum computing. Current approaches in solid-state quantum technology are based on brute-force methods, where each and every qubit requires at least one unique control line—an approach that will become unsustainable when scaling to the required millions of qubits. Here, inspired by random-access architectures in classical electronics, we introduce the shared control of semiconductor quantum dots to efficiently operate a two-dimensional crossbar array in planar germanium. We tune the entire array, comprising 16 quantum dots, to the few-hole regime. We then confine an odd number of holes in each site to isolate an unpaired spin per dot. Moving forward, we demonstrate on a vertical and a horizontal double quantum dot a method for the selective control of the interdot coupling and achieve a tunnel coupling tunability over more than 10 GHz. The operation of a quantum electronic device with fewer control terminals than tunable experimental parameters represents a compelling step forward in the construction of scalable quantum technology.

## Abstract (web summary)

An efficient control strategy is designed for quantum dot arrays, drawing inspiration from classical semiconductor technology. A two-dimensional array of 16 semiconductor quantum dots is operated using only a few shared control lines.

## Main

Fault-tolerant quantum computers will require millions of interacting qubits^1–3^. Scaling to such extreme numbers imposes stringent conditions on all the hardware and software components, including their integration^4^. In semiconductor technology, decades of advancements have led to the integration of billions of transistor components on a single chip. A key enabler has been the ability to control such a large number of components with only a few hundred to a few thousand control lines^5,6^. In quantum technology, such a game-changing strategy has yet to be embraced owing to the fact that qubits are not sufficiently similar to each other. Nowadays, leading efforts in the solid state, such as superconducting and semiconducting qubits, require that each and every qubit component is connected to at least one unique control line^7^. Clearly, this brute-force approach is not sustainable for attaining practical quantum computation.

The development of spin qubits in semiconductor quantum dots has been strongly inspired by classical semiconductor technology^8–10^. Advanced semiconductor qubit systems are based on complementary-metal–oxide–semiconductor-compatible materials and even foundry manufactured qubits have been realized^11,12^. In addition, it is anticipated that the small qubit footprint and compatibility with (cryo-)complementary metal–oxide–semiconductor electronics will open up avenues to build integrated quantum circuits^13,14^. To enable the efficient control of large qubit architectures with a sustainable number of control lines, proposals of architectures inspired by classical random-access systems have been put forward^15,16^. However, their practical realization has been, so far, prevented by device quality and material uniformity.

Here we take the first step towards the sustainable control of large quantum processors by operating semiconductor quantum dots in a crossbar architecture. This strategy enables the manipulation of the most extensive semiconductor quantum device with only a few shared control terminals. This is accomplished by exploiting the high quality and uniformity of strained germanium quantum wells^17^, by introducing an elegant gate layout based on diagonal plunger lines and double-barrier gates, as well as by establishing a method that directly maps the transitions lines of charge stability diagrams to the associated quantum dots in the grid. We operate a two-dimensional 16-quantum-dot system and demonstrate the tune up of the full device to the few-hole regime. In this configuration, we also prove the ability to prepare all the quantum dots in the odd-charge occupation, as a key step for the confinement of an unpaired spin in each site^18,19^. We then introduce a random-access method for addressing the interdot tunnel coupling and find a remarkable agreement in the response of two vertically and horizontally coupled quantum dot pairs. We also discuss some critical challenges to efficiently operate future large quantum circuits.

## A two-dimensional quantum dot crossbar array

**Fig. 1:**
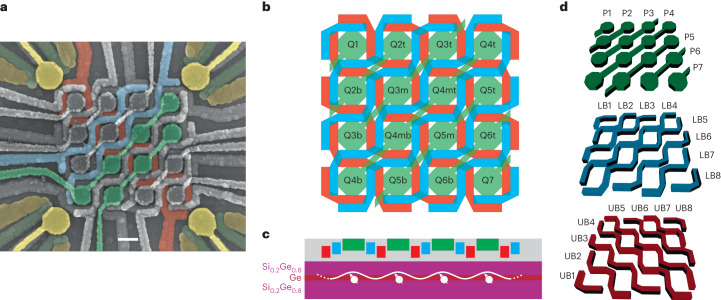
A 16 quantum dot crossbar array. **a**, False-colour scanning electron microscopy image of the crossbar array device. The architecture consists of two staircase barrier gate layers (two lines of each layer are shown in red and blue), and one plunger gate layer (two lines are illustrated in green). Here 16 quantum dots are defined under the plungers, whereas four charge sensors in the form of single-hole transistors are located at the corners (ohmic contacts in orange, barriers in dark green and plungers in yellow). The scale bar is 100 nm (which is also the diameter of the designed plunger gate). This shared-terminal control approach enables to control a number of quantum dots (*g*) with a sublinear number of control terminals (*T*). Here the scaling is given by *T* = 6*g*^1/2^ − 1 (Supplementary Fig. 1). **b**, Schematic of the device and labelling of each quantum dot. We choose to label the quantum dots after their positions on their controlling plunger line, for example, the quantum dot Q6b(t) is located on the bottom (top) site controlled by plunger line P6. **c**, Schematic of the device cross section. Holes are isolated in quantum dots in a 55-nm-deep germanium quantum well in a silicon–germanium heterostructure grown on a silicon wafer. **d**, Shared control elements: from the bottom of the gate stack, eight UB barrier gates, eight LB barrier gates and seven P plunger gates. The overlay of these layers is visible in **b**.

Our shared-terminal control approach for a two-dimensional quantum dot array with dots Q1, Q2t…Q7 is based on a multilayer gate architecture (Fig. 1a–d). We use two barrier layers (with gates UB*i* and LB*i*, where *i* ∈ [1, 8]) to control the interdot tunnel couplings, and exploit a layer of plunger lines (P*i*, where *i* ∈ [1, 7]) to vary the on-site energies (Fig. 1d). In contrast to brute-force implementations, here a single plunger gate is employed to control up to four quantum dots, and an individual barrier up to six nearest-neighbour interactions. In analogy with classical integrated circuits, this strategy enables to manage a number of experimental parameters (that is, quantum dot energies and interdot couplings) with a sublinear number of control terminals, an approach that may overcome, among other aspects, the wiring interconnect bottleneck of large-scale spin qubit arrays (Supplementary Fig. 1)^6,7^.

To monitor the quantum dot array, we make use of charge-sensing techniques^18^. Four single-hole transistors at the corners of the array (named after their cardinal positions: NW as northwest, NE as northeast, SW as southwest and SE as southeast) act as charge sensors as well as hole reservoirs for the array (Supplementary Fig. 2). The simultaneous readout of their electrical response in combination with fast rastering pulse schemes enables us to continuously measure the two-dimensional charge stability diagrams in real time (that is, in the video-mode technique), and updating the direct-current (d.c.) gate voltages controlling the array^20,21^.

To bring the device in the 16-quantum-dot configuration, we identify an alternative strategy to the tune-up methods established for individually controlled quantum dot devices^22^. We begin by lowering all the gate voltages to their starting values based on previous experiments and by defining a set of virtual gates as linear combinations of real gates^22–24^. Such virtual gates are defined to eliminate crosstalk with the charge sensors and to independently control the on-site energies^23,24^ (Supplementary Fig. 3). Here we will refer to vP*i* as the virtual gate associated to the actual gate P*i*.

We continue the tuning of the device by adjusting the gates controlling the quantum dots at the corner (that is, those closest to the charge sensors) until we accumulate the first few holes as signalled by the first addition lines in the charge stability diagrams. We then proceed with the tune up of the adjacent dots and finish with the quantum dots furthest to the sensor. Owing to the homogeneity of our heterostructure^25^ and the symmetry of our gate layout, the accumulation of the first few holes in the quantum dots controlled by the same plungers occurs at similar gate voltages.

Rather, challenges in tuning up the array are mainly due to elements outside the array. In fact, we observe that small variations in gate voltages impact the electrostatics of the dense gate fanout area, which, in turn, affects the charge sensors and readout quality. Furthermore, specific gate settings cause unintentional quantum dots under the gate fanout, which restrict the operational window. Altogether, these issues are a challenge for the implementation of automated tuning methods^26^, but we envision that the integration of a lower layer of ‘screening’ gates or the implementation of the gate fanout in the third dimension^27^ can mitigate this issue (Supplementary Note 4).

## From multidot charge stability diagrams to quantum dot identification

**Fig. 2:**
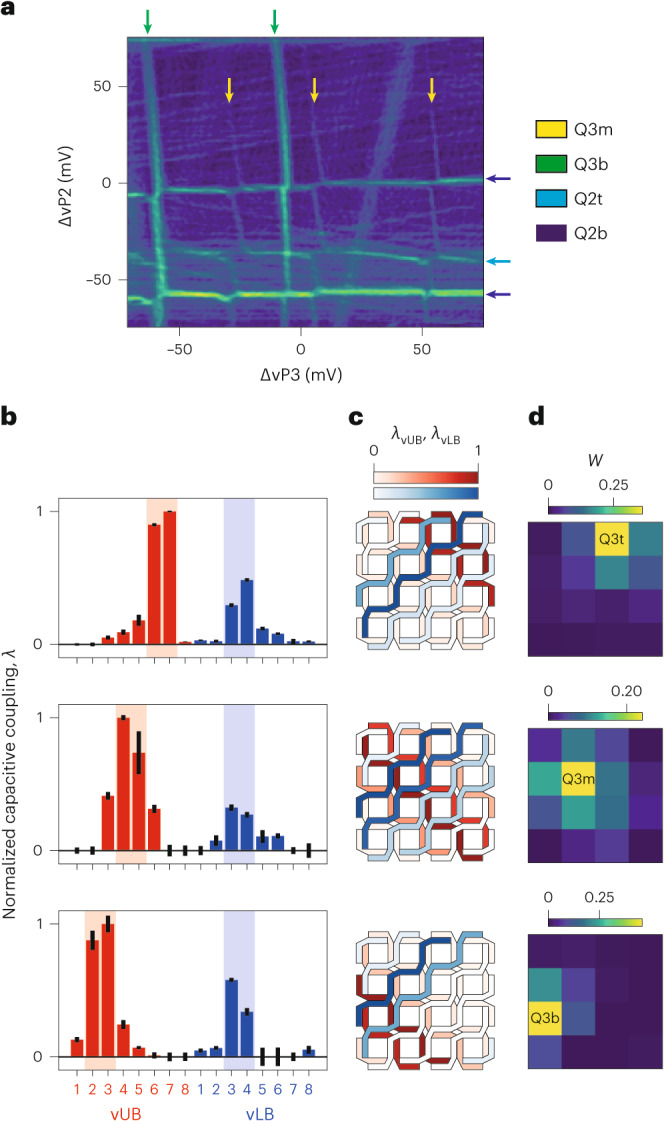
From multidot charge stability diagrams to quantum dot identification. **a**, Few-hole charge stability diagram obtained by combining the signal gradients of the SW and NW charge sensors. The labels indicate the corresponding quantum dot for each addition line. At (0, 0), the fillings of Q2t, Q2b, Q3t, Q3m and Q3t are 0, 0, 0, 3 and 0, respectively. **b**, Histograms of the (normalized) capacitive couplings *λ* of the barrier gates to the transition lines (red for vUB and blue for vLB virtual gates) obtained by analysing three different sets of transition line coupled to virtual plunger vP3. The middle and bottom panels are extracted from the shift in the transition lines at ~(–20, –63) and (–15, –7) in **a**, respectively. The top panel (relative to dot Q3t) is obtained from a measurement shown in Supplementary Fig. 14g,h, where the transition line is more visible. The red and blue backgrounds are added to emphasize the two barriers with the highest couplings. The data points correspond to the peak of the chart bars as well as the centres of the error bars. Each error bar is the standard deviation of the parameter obtained from the linear fit. **c**, Device layout with the capacitive couplings colour coded on the filling of the gate lines. Here both vUB and vLB capacitive couplings, namely, *λ*_vUB_ and *λ*_vLB_, respectively, are normalized to their maximum values. **d**, Visualization of the probability (*W*) calculated from the shift in the three sets of addition lines (Methods). A comparison of the top, middle and bottom panels of **b**–**d** clearly distinguishes the three Q3 quantum dots.

Moving forward, a direct result of our control approach is the fact that upon sweeping the voltage of a plunger gate controlling *n* quantum dots, up to *n* sets of charge transitions can be observed, each associated with (un)loading an additional hole in one of the *n* quantum dots. For the case of vP2 and vP3, this results in a charge stability diagram (Fig. 2a) where a number of vertical and horizontal charge addition lines marking well-separated charge states are visible. However, because of our control approach, a priori it is unknown to which of the Q3 (Q2) quantum dots these vertical (horizontal) lines are associated.

Here we solve this problem by establishing a statistical method that maps such transition lines to the respective quantum dot. In our protocol, we first evaluate the shift in the charge transition lines induced by a voltage variation in each barrier gate to estimate the (normalized) capacitive coupling *λ* between each barrier gate and the associated quantum dot (Fig. 2b,c). Because the two barrier layers form a grid of lines and columns, we can use their capacitive couplings to infer the spatial location of the quantum dot in the array. For this purpose, we consider the normalized capacitive couplings of the two orthogonal barrier sets, namely *λ*_vUB_ and *λ*_vLB_, as two independent probability distributions. We then use *λ*_vUB_ and *λ*_vLB_ to calculate the combined probability *W* on each of the 16 sites (Fig. 2d and Methods). Finally, our protocol ends assigning the site with the maximum probability to the quantum dot loaded via the specific charge addition lines. In practice, *W* quantifies how much an electric field generated on each site is perceived by a hole in a specific quantum dot site. Hence, a low (high) *W* value identifies a location that is weakly (strongly) coupled to the analysed quantum dot.

In Fig. 2b–d, we show how this routine is effective in distinguishing and characterizing the three Q3 quantum dots, whereas similar results are obtained for the remaining dots in the grid (Supplementary Figs. 4–6). The demonstrated ability of labelling multiple quantum dot charge stability diagrams makes this method an important tool in the tune up of large quantum dot devices.

## Quantum dot occupancies

Although useful in drastically reducing the number of control terminals, a crossbar approach is effective for spin-based quantum computing if it enables to isolate a single or unpaired spin in the individual quantum dots^8,16^. Here we demonstrate the tune up of the array to an odd-charge-occupancy configuration with 11 quantum dots filled with one hole and five quantum dots filled with three holes.

Our setup allows for fast charge stability maps, although of a size that it is often insufficient to fully visualize the absence of further transition lines in the zero-charge state. Therefore, we perform repeated scans of the kind ΔvP*x* versus ΔvP*y*, and increasing the d.c. voltage vP*x* in discrete steps of 10 mV. We stop the sequence when the quantum dot controlled by vP*x*, say Q*x*, is fully depleted. We present these emptying sequences in Supplementary Videos 1–12 and describe them in Supplementary Note 7, where we use addition lines or interdot transitions to monitor the charge occupancy.

In general, multidot transition lines, low charge sensitivity, spurious quantum dots and low reservoir tunnel coupling may complicate the assessment of occupancy. In fact, quantum dots that are located in the core of the array are loaded/unloaded by means of co-tunnelling processes via the outer dots^28^, leading to latching transition lines and elongated charge interdot transitions when the reservoir-dot tunnelling time approaches the timescale of our scan (~0.01 s) (refs. ^29–32^). We note that adding reservoirs within the array may reduce this effect and simplify the tune up. However, optimal qubit operation and high qubit connectivity may require low tunnel coupling between the qubits and reservoirs. Thus, initializing the array without having each quantum dot strongly coupled to a reservoir is highly relevant^6,10,33^.

**Fig. 3:**
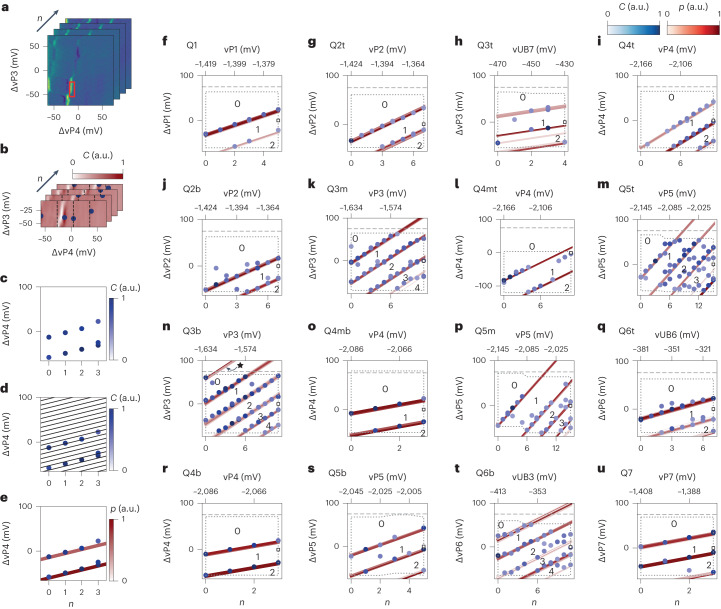
Counting holes in a 16 quantum dot array. **a**–**e**, Illustration of the detection algorithm on the Q4b charge transition lines. From the charge stability diagrams (**a**) labelled with index *n*, we compute the image correlation (*C*) maps (**b**), with the reference feature in the red box in **a** containing a portion of a Q4b charge transition. After splitting the correlation maps in four vertical sections (dashed lines in **b**), we determine the local maxima in each of them (blue markers in **b**). We track the thresholded local maxima across all the scans of the sequence (**c**). We quantify the occurrence of high-correlation features along potential lines with a fixed expected slope and varying intercepts (black lines in **d**). In **d**, we show only one-fifth of the actual sampling lines for clarity. In **e**, we colour code all the potential lines according to their associated *p* value. Emerging peaks in *p* (that is, red traces) identify the actual first two Q4b charge transitions. **f**–**u**, Same data as **e**, but for all the 16 quantum dots. The numbers in each plot indicate the quantum dot fillings, according to the algorithm. In three cases (Q3t, Q6b and Q6t) out of the 16, we step a barrier, rather than a plunger, to better isolate the shift of only one of the dots under the same plunger line. High-correlation features (blue points) are found up to the bounds of the area of the datasets analysed by the protocol, highlighted by the dotted grey polygon. The horizontal dashed line indicates the actual limit of the dataset. The star in **n** indicates a false-positive transition line.

To track specific transition lines in complex multidot charge stability diagrams, we have defined an algorithm based on an image correlation analysis, which may be refined by machine learning methods^26,34–36^. First, we select a small window from a specific charge stability diagram in the sequence containing a Q*x* charge addition line. We refer to this as the Q*x* reference feature (Fig. 3a) for Q4b. Second, we compute digital image correlation (*C*) maps of all the charge stability diagrams in the sequence with respect to the previously defined reference feature (Supplementary Note 8). Then, we divide the correlation maps into four sections (delimited by vertical dashed lines, Fig. 3b), with a size that is smaller than the typical addition voltage. This choice results in a high probability of having, at most, a single occurrence of a Q*x* charge transition per section. We then select the coordinates of the points with the maximum correlation in each section, and threshold them to ensure that only points with high correlation are passed.

Because of the d.c. voltage step between each map, the same high-correlation feature, if reproducible, is then expected to be found at a ΔvP*x* value that is 10 mV higher than in the previous charge stability diagram (Fig. 3c). Therefore, in a plot of coordinates with high correlation versus scan index (*n*), we expect these points to follow a line with a well-defined slope of 10 mV per scan. To assess the presence of possible charge transition lines within the clouds of high-correlation coordinates, we consider a series of potential lines with a slope of 10 mV per scan and varying intercepts (Fig. 3d). We then define quantity *p* that accounts for the density of high-correlation points falling along each line. In practice, *p* represents a quantum dot transition-line likelihood (Fig. 3e); Supplementary Note 8 provides details on its mathematical description.

Figure 3f–u presents the detected coordinates with high correlation of all the 16 quantum dots as a function of the scan index and associated stepped voltage, together with all the possible transition lines plotted with a colour that is proportional to their respective *p* value. High values of *p* are rendered as visible lines intercepting several high-correlation features, enabling a rapid visualization of the charge transition lines of all the quantum dots of the array.

By comparing all the panels in this figure, we can resolve a small shared gate-voltage window around the tuning point defined by ΔvP*x* = 0 at the largest *n* where all the quantum dots are in the odd-charge regime (Supplementary Fig. 8).

Although the algorithm is successful in detecting the transition lines of every quantum dot, we note that it returns a false-positive line for the case of Q3b. The first visible Q3b transition (Fig. 3n, star) is, in reality, an outlier due to a detected feature at *n* = 0 and ΔvP3 ≈ 55 mV not related to Q3b but due to an artefact of a charge sensor (Supplementary Note 7). Even though the algorithm is remarkably strong in detecting transitions, a definite conclusion on the odd occupancy is most probably only achieved by performing a coherent operation on each qubit.

## Interdot coupling control

The ability to selectively tune the interdot coupling in a quantum dot architecture is crucial for generating exchange-based entanglement between the semiconductor qubits^8^. Here, inspired by the wordlines and bitlines approach as in dynamic random-access memories^13,16^, we exploit the double-barrier design to spatially define and activate unique points in the grid structure. Conceptually, each two-barrier intersection point can be set by the respective voltages in the following four configurations: (ON, ON), (ON, OFF), (OFF, ON) and (OFF, OFF). For selective two-qubit operations in qubit arrays, the voltage set points should be calibrated such that only when both barriers are in the ON state, a two-qubit interaction is activated, leaving all the other pairs non-interacting (Supplementary Fig. 9).

**Fig. 4:**
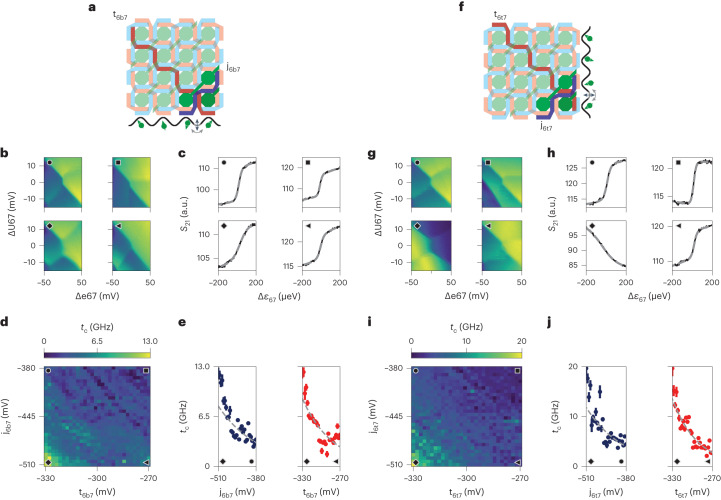
Addressable control of the interdot tunnel coupling using double-barrier gates. **a**,**f**, Schematic of the crossbar indicating the two intersecting barriers (red and blue) controlling the Q6b–Q7 (**a**) and Q6t–Q7 (**f**) interdot tunnel couplings. **b**,**g**, Exemplary charge stability diagrams taken via reflectometry methods showing the (Q6b, Q7) (**b**) and (Q6t, Q7) (**g**) charge states at four different virtual barrier-voltage configurations near the (3,1)–(2,2) transition. At the centre of the panels, the vertical interdot transition line is clearly visible. The circle, square, diamond and triangle markers correlate each map to the voltages of (t_6b7_, j_6b7_) = (t_6t7_, j_6t7_) = −(330, 380), (270, 380), (330, 510) and (270, 510) mV, respectively. **c**,**h**, Charge polarization traces (black) relative to **b** (**c**) and **g** (**h**), together with the best fit (dashed grey). **d**,**i**, Colour maps of the two-axis controlled tunnel couplings of the Q6b–Q7 (**d**) and Q6t–Q7 (**i**) systems, with markers located at the respective voltages. We note that a variation in the virtual barrier corresponds to the same variation in the real gate. **e**,**j**, Vertical (left panel) and horizontal (right panel) linecuts of **d** and **i** at t_6b7_ = t_6t7_ = −330 mV (**e**) and j_6b7_ = j_6t7_ = −510 mV (**j**). The grey traces are fits with an exponential function of the data, from which we obtain the four effective barrier lever arms (Methods).

Here we implement a proof of principle of this method by demonstrating the two-barrier control of the interdot tunnel coupling in a horizontal and vertical pair of quantum dots. To this end, we investigate the tunnel coupling variations of the horizontal Q6b–Q7 and vertical Q6t–Q7 quantum dot pairs as a function of the two intersecting barriers. Starting from the respective UB4 and LB7 gates, we define the virtual barriers t_6b7_ and j_6b7_ (Methods), which separate the quantum dots Q6b and Q7, and keeping their detuning (e67) and on-site (U67) voltages constant at the (3,1)–(2,2) Q6b–Q7 interdot transition (Fig. 4a). After obtaining the detuning lever arm ${\alpha }_{{\epsilon }_{67}}$ to convert the detuning voltage into an energy scale ($\Delta {\epsilon }_{67}={\alpha }_{{\epsilon }_{67}}\times \Delta {{{\rm{e}}}}67$), we evaluate the strength of the interdot couplings by analysing the charge polarization lines along the detuning axis at ΔU67 = 0 (Fig. 4b,c)^37^. By systematically performing this measurement, we demonstrate that the tunnel coupling can be effectively controlled by both barriers (Fig. 4d,e). At values of (t_6b7_, j_6b7_) = (−270, −380) mV, the coupling is virtually OFF, but our method is inherently not accurate for *t*_c_ ≤ 3 GHz, because thermal energy dominates the broadening of the polarization line^31,37^. In contrast, on activating both barriers at (t_6b7_, j_6b7_) = (−330, −510) mV, the tunnel coupling is turned on, approximately following an exponential trend (Methods). Within the displayed voltage range, we can tune it well above 10 GHz, much higher than in the configuration in which only one barrier is activated. We perform the same experiment on the dot pair Q6t–Q7 by defining virtual barriers t_6t7_ and j_6t7_ based on gates UB5 and LB7, respectively (Fig. 4f and Methods). Using the same barrier-voltage window as for Q6b–Q7, we find that the coupling tunability of the pair Q6t–Q7 is comparable with the previous pair, with a virtually OFF state (≤3 GHz) at (t_6t7_, j_6t7_) = (−270, −380) mV, and with an ON state reaching 20 GHz at (t_6t7_, j_6t7_) = (−330, −510) mV (Fig. 4g–j). These results are corroborated by the two-barrier tunability of the interdot capacitive coupling for both double-dot systems (Supplementary Fig. 10) and by the tunability of another dot pair (Supplementary Fig. 11). We envision that for rapid qubit exchange operations at the charge symmetry point, the required barrier-voltage window might be different from our measurable window via polarization lines. Specifically, for state-of-the-art values of ON (OFF) exchange interaction of 50 MHz (10 kHz) and a typical charging energy of 1 mV, the required tunnel coupling is ~2.00 (0.02) GHz, which can be better calibrated via qubit spectroscopy techniques^20,38,39^.

## Conclusions

By implementing a strategy that allows to address a large number of quantum dots with a small number of control lines, we have operated the most extensive two-dimensional quantum dot array so far, to the best of our knowledge. With this approach, the number of gate layers is independent of the grid size, which greatly simplifies the nanofabrication of quantum dot arrays. With the introduction of a double-barrier paradigm and a statistical method for labelling multidot charge stability diagrams, we have demonstrated two critical requirements for quantum logic in shared control architectures: the tunability of 16 interacting quantum dots into an odd-charge state with an unpaired spin and the proof of principle of a method for the selective control of interdot tunnel coupling, which is crucial for the control of the exchange interaction. We envision that future crossbar arrays may find applications in large and dense two-dimensional quantum processors or as registers that are coupled via long-range quantum links for networked computing.

## Methods

### Fabrication

The device is fabricated on a Ge/SiGe heterostructure where a 16-nm-thick germanium quantum well with a maximum hole mobility of 2.5 × 10^5^ cm^2^ V^−1^ s^−1^ is buried 55 nm below the semiconductor/oxide interface^25,40^. We design the quantum dot plunger gates with a diameter of 100 nm, and the barrier gates separating the quantum dots with a width of 30 nm. The fabrication of the device follows these main steps. First, 30-nm-thick Pt ohmic contacts are patterned via electron-beam lithography, evaporated and diffused in the heterostructure following an etching step to remove the oxidized Si cap layer^41,42^. A three-layer gate stack is then fabricated by alternating the atomic layer deposition of an Al_2_O_3_ dielectric film (with thicknesses of 7, 5 and 5 nm) and the evaporation of Ti/Pd metallic gates (with thicknesses of 3/17, 3/27 and 3/27 for each deposition, respectively). After dicing, a chip hosting a single crossbar array is then mounted and wire bonded on a printed circuit board. Before cool down in a dilution refrigerator, we tested two nominally identical crossbar devices in a 4 K helium bath as per the screening procedure^38^. Both devices exhibited the functionality of full gates and ohmic contacts, and one of them was mounted in a dilution refrigerator.

### Experimental setup

The experiment is performed in a Bluefors dilution refrigerator with a base temperature of 10 mK. From a Coulomb peak analysis, we extract an electron temperature of 138 ± 9 mK, which we use to estimate the detuning lever arm (Supplementary Figs. 12 and 13). We utilize an in-house built battery-powered SPI rack (https://qtwork.tudelft.nl/~mtiggelman/spi-rack/chassis.html) to set the d.c. voltages, whereas we use a Keysight M3202A arbitrary waveform generator (AWG) to apply alternating-current rastering pulses via coaxial lines. The d.c. and alternating-current voltage signals are combined on the printed circuit board with bias-tees and applied to the gates. Each charge sensor is galvanically connected to a NbTiN inductor with an inductance of a few microhenries, forming a resonant tank circuit with resonance frequencies of ~100 MHz. In our experiment, we have observed only three out of four resonances, probably due to a defective inductor. Moreover, because the two resonances overlap substantially, we mostly avoid using reflectometry (unless explicitly stated in the text) and use fast d.c. measurements with a bandwidth of up to 50 kHz. The four d.c. sensor currents are converted into voltages, amplified and simultaneously read by a four-channel Keysight M3102A digitizer module with 500 megasamples s^–1^. The digitizer module and several AWG modules are integrated into a Keysight M9019A peripheral component interconnect express extensions for the instrumentation chassis. Charge stability diagrams here typically consist of a 150 × 150 pixel scan with a measurement time per pixel of 50 μs. Throughout this Article, we refer to Δg*i* to identify a ramp supplied by an AWG to the gate g*i* with respect to a fixed d.c. reference voltage. To enhance the signal-to-noise ratio, we average the same map 5–50 times, obtaining a high-quality map within a minute.

### Tune-up details

Throughout the experiment, we have tuned all the 16 quantum dots of the device two times. In the first run, the gate voltages were optimized to minimize the number of unintentional quantum dots to better visualize and characterize the crossbar quantum dots (Fig. 2 and Supplementary Fig. 14). In the second run, the stray dots were neglected to tune the quantum dot array into a global odd-occupation regime (Fig. 3). Between the two tune-up cycles, the gate voltages were reset to zero without thermally cycling the device. The protocol followed in the two tuning procedures was the same, but the need for emptying accidental quantum dots in the first session led to some restrictions in the voltage window of certain gates. The starting gate-voltage values for the tune up are –300 mV for barriers and –600 mV for plungers. In Supplementary Fig. 15, we display the d.c. gate voltages relative to the measurements displayed in Fig. 3, with the crossbar array tuned in the odd-charge occupation. In this regime, we also study the variability in the first hole voltage onset in each dot, obtaining −1,660 ± 290 mV (Supplementary Fig. 16). Furthermore, we characterize the variability in the transition-line spacing to be ~10–20% as a metric for the level of homogeneity of the array (Supplementary Fig. 17)^43^. Supplementary Note 4 discusses strategies to further reduce these variations.

The odd-charge occupancy is demonstrated by emptying each quantum dot (Supplementary Videos 1–12). All the datasets underlying Fig. 3 and Supplementary Videos 1–12 are taken at the same gate-voltage configuration on the same day. Still, across all the maps, there are minimal voltage differences, the largest being a variation of 6 mV in vP1 that, however, does not affect the Q1, Q2b and Q2t occupancies (Supplementary Table 1). During the experiment, gate UB8 did not function properly, possibly due to a broken lead. To compensate for this effect and to enable charge loading in P3t and P5t dots, we set UB7 at a lower voltage compared with the other UB gates. Additionally, LB1 is set at a comparatively higher voltage to mitigate the formation of accidental quantum dots under the fanout of LB1 and P1 at lower voltages. The first addition line of such an accidental quantum dot is visible as a weakly interacting horizontal line (Fig. 3a).

### Virtual matrix

The matrix *M* defined by $\bf{\overrightarrow{G}}=M \times \bf{\overrightarrow{{{{\rm{v}}}}G}}$, with virtual gates $\overrightarrow{{\rm{v}}\bf{G}}$ and actual gates $\overrightarrow{\bf{G}}$ is shown as a colour map in Supplementary Fig. 3. For the tunnel coupling experiments presented in Fig. 4, we employ additional virtual-gate systems for achieving independent control of detuning voltages e67 and U67, as well as the interdot interactions via virtual barriers t_6b7_, j_6b7_, t_6t7_ and j_6t7_. With SE_P defined as the SE plunger gate, we write

$$\begin{array}{rcl}\left(\begin{array}{c}\,{{\mbox{P5}}}\,\\ \,{{\mbox{P6}}}\,\\ \,{{\mbox{P7}}}\,\\ \,{{\mbox{SE\_P}}}\,\end{array}\right)&=&\left(\begin{array}{cc}0.04&-1.2\\ -0.5&0.9\\ 0.492&0.9\\ -0.08&-0.26\end{array}\right)\left(\begin{array}{c}\,{{\mbox{e67}}}\,\\ \,{{\mbox{U67}}}\,\end{array}\right)\\ \left(\begin{array}{c}\,{{\mbox{P6}}}\,\\ \,{{\mbox{P7}}}\,\\ \,{{\mbox{UB5}}}\,\\ \,{{\mbox{LB7}}}\,\\ \,{{\mbox{SE\_P}}}\,\end{array}\right)&=&\left(\begin{array}{cc}-1.28&-0.33\\ -1.18&-0.72\\ 1&0\\ 0&1\\ 0.15&-0.01\end{array}\right)\left(\begin{array}{c}{{{{\rm{t}}}}}_{6{{{\rm{t}}}}7}\\ {{{{\rm{j}}}}}_{6{{{\rm{t}}}}7}\end{array}\right)\\ \left(\begin{array}{c}\,{{\mbox{P6}}}\,\\ \,{{\mbox{P7}}}\,\\ \,{{\mbox{UB4}}}\,\\ \,{{\mbox{LB7}}}\,\\ \,{{\mbox{SE\_P}}}\,\end{array}\right)&=&\left(\begin{array}{cc}-2.05&-0.97\\ -1.18&-0.41\\ 1&0\\ 0&1\\ -0.19&-0.01\end{array}\right)\left(\begin{array}{c}{{{{\rm{t}}}}}_{6{{{\rm{b}}}}7}\\ {{{{\rm{j}}}}}_{6{{{\rm{b}}}}7}\end{array}\right)\end{array}.$$

### Quantum dot identification

To obtain the capacitive coupling of all the barrier gates to a set of transition lines (Fig. 2b), we acquire and analyse sets of 112 charge stability diagrams. The same charge stability diagram is taken after stepping each barrier gate around its current voltage in steps of 1 mV in the range of –3 to 3 mV (that is, 7 scans × 16 barriers). The number of charge stability diagrams required to identify all the quantum dots scales linearly with their total number. The number of maps results from the product of the number of plungers and barrier gates, both of which scale as its square root. We emphasize that an array with individual control would also require a linear number of charge stability diagrams to infer each dot. In the analysis, we first subtract a slowly varying background to the data (with the ndimage.gaussian.filter function of the open-source SciPy package version 1.7.1) and then calculate the gradient of the map (with the ndimage.gaussian_gradient_magnitude function). For a given linecut of such two-dimensional maps, we extract the peak position using a Gaussian fit function. Due to cross-capacitance, the transition-line positions manifest a linear dependence on each of the 16 barriers, which we quantify by extracting the linear slope (Supplementary Fig. 4). After normalization to the maximum value, these parameters are named capacitive couplings (*λ*) and because of the grid structure of the two barrier layers, the first information of where the hole is added/removed to/from is obtained. To extract the quantum dot positions, we consider the capacitive couplings to the vUB (*λ*_vUB_) and vBL (*λ*_vLB_) gates as two independent probability distributions. With this approach, the integral of *λ*_vUB_ (*λ*_vLB_) between vUB*i* (vLB*k*) and vUB*j* (vLB*l*) returns a ‘probability’ *p*_U,(*i*,*j*)_ (*p*_L,(*k*,*l*)_) to find the dot between these control lines. As a result, the combined probability in the site confined by these four barriers is given by the product of these elements: *w*_(*i*,*j*),(*k*,*l*)_ = *p*_U,(*i*,*j*)_ × *p*_L,(*k*,*l*)_. We note that the sum of the 16 probabilities returns 1. As already observed in another work^32^, the gates cross-coupling to a specific quantum dot defined in a germanium quantum well manifest a slow falloff in space (that is, gates with a distance to the dot of >100 nm still have a considerable cross-coupling to the dot). This can be attributed to the rather large vertical distance between the gates and quantum dots (>60 nm), and is in contrast with experiments in Silicon–metal–oxide–semiconductor devices where the falloff is rather immediate due to tight charge confinement. This aspect explains why our probability *W* at the identified quantum dot reaches a maximum of 0.25−0.50.

### Tunnel coupling evaluation

For the estimation of the tunnel coupling results presented in Fig. 4, we established an automated measurement procedure that follows this sequence: (1) we step the virtual barriers across the two-dimensional map (*t*, *j*); (2) at each barrier configuration, we take a two-dimensional (e67, U67) charge stability map (Fig. 4b–g); (3) we identify the accurate position of charge interdot via a fitting procedure of the map (Supplementary Fig. 10)^44^; (4) we perform small adjustments at the e67 and U67 virtual gates to centre the interdot at the (0, 0) d.c. offset; (5) we measure the polarization line by using ~0.1 kHz AWG ramps (Fig. 4c,h). For an accurate analysis, each polarization line is the result of an average of 150 traces, using a measurement integration time of 50 μs per pixel. With this method, the full 30 × 30 maps are taken in a few hours. We fit the traces considering an electron temperature of 138 mK and a detuning lever arm of ${\alpha }_{{\epsilon }_{67}}$ = 0.012(4) eV V^–1^, extracted from a thermally broadened polarization line (Supplementary Fig. 13). We observe that the extracted tunnel coupling approximately follows an exponential trend as a function of barrier gates. We fit the data presented in Fig. 4e,j with the $A\times {\rm{e}}^{-B{V}_{\rm{g}}}$ function, where *A* is a prefactor, *B* is the effective barrier lever arm and *V*_g_ is the gate axis. We find that the effective barrier lever arms of *j*_6b7_ and *t*_6b7_ are 0.007 ± 0.002 and 0.021 ± 0.003 mV^−1^, respectively. Similarly, *j*_6t7_ and *t*_6t7_ are 0.008 ± 0.001 and 0.026 ± 0.003 mV^−1^, respectively. This indicates that the real barrier LB7 controls the vertical and horizontal couplings in a similar manner. Altogether, these results indicate that the lower barrier layer of UB gates is ~3 times more effective than the upper barrier layer of LB gates. This is consistent with what is found in Fig. 2b and Supplementary Fig. 5. We note that for qubit operations in such a crossbar array, it is actually necessary to fully characterize and calibrate the two-barrier tunability of all the 24 nearest neighbours. Performing this task requires improving our hardware implementation further and is beyond the scope of this work.

## Online content

Any methods, additional references, Nature Portfolio reporting summaries, source data, extended data, supplementary information, acknowledgements, peer review information; details of author contributions and competing interests; and statements of data and code availability are available at 10.1038/s41565-023-01491-3.

### Supplementary information

Supplementary Information Supplementary Figs. 1–17, Tables 1–4 and Notes 1–16.

Supplementary Video 1 Sequence of measurements (for improved visibility, we show the raw data after subtracting a slow-varying background) that demonstrates the presence of a single hole in quantum dot Q1. We use a dashed guide-for-the-eye trace to indicate the first two Q1 horizontal addition lines. The faint vertical transition lines are Q2t and Q2b addition lines.

Supplementary Video 2 Sequence of measurements (for improved visibility, we show the raw data after subtracting a slow-varying background) that demonstrates the single-hole occupancy of both Q2b and Q2t. We use two sets of dashed guide-for-the-eye traces to indicate the first two Q2b and Q2t transition lines. The two rightmost vertical lines are Q1 transition lines, whereas the left one at ΔvP1 ≈ −50 mV (indicated with a dashed vertical line) is associated to an accidental dot outside the array, formed under the fanout of gates P1 and LB1.

Supplementary Video 3 Sequence of charge stability diagrams (for improved visibility, we display the partial derivative of the raw data along ΔvP3) demonstrating the third-hole occupancy of Q3b. The prominent horizontal lines are Q3b transition lines, indicated with dashed guide-for-the-eye traces. In the first two scans, the two bright features located at (30, 60) and (26, 46) are due to artefacts of the charge sensors, possibly due to a transmission resonance between the charge sensors in the fanout. We note that these features move in the opposite direction than the quantum dot lines. The three quasi-vertical lines (one is indicated with a dashed black line) are attributed to a quantum dot located under the fanout of P2 (Supplementary Note 6).

Supplementary Video 4 Sequence of charge stability diagrams (for improved visibility, we show the raw data after subtracting a slow-varying background) demonstrating the third-hole occupancy of Q3m. In the first two scans, the vertical dark feature located at ΔvP2 ≈ 20 mV is due to an artefact of the charge sensors. The vertical lines are Q2s transition lines. The lines associated to Q2b appear with a positive slope near the charge interdots due to latching effects. Supplementary Fig. 7 discusses these features in detail.

Supplementary Video 5 Sequence of charge stability diagrams (for improved visibility, we show a linear combination of the derivatives along the ΔvP4 axis of the SW and SE charge sensors) demonstrating the single-hole occupancy of Q3t. In the maps, Q3t is visualized via the indicated charge interdots (grey squares) with Q4t quantum dot (vertical dark lines). To isolate the response of Q3t from the other two Q3 quantum dots, we step the barrier gate vUB7, which couples much less to Q3b and Q3t. The predominant vertical transition lines are associated to Q4b and Q4t, whereas the most visible horizontal lines are the Q3b transition lines that display charge interdots with Q4b.

Supplementary Video 6 Sequence of charge stability diagrams (we display the raw data after subtracting a slow-varying background) demonstrating the single-hole occupancy of Q4b and Q4mb, whose transition lines are quasi-parallel and very close to each other in gate voltage. The horizontal lines are Q3b transition lines.

Supplementary Video 7 Sequence of measurements (we show the raw data after subtracting a slow-varying background) demonstrating the single-hole occupancy of Q4mt and Q4t. Different from Q4t, the transition lines of Q4mt have a positive slope in the virtual axis framework, which is also accentuated by the slow tunnel time to the reservoir (latching effects), indicating a variability in the cross-capacitance to the dots controlled by P4, possibly due to disorder. The assessment of the identified transition lines with quantum dot Q4mt was performed in a regime with higher tunnel coupling to the reservoir, from the cross-capacitance of the nearby barrier gates (Fig. 2). The horizontal lines are associated to all the three Q5 quantum dots.

Supplementary Video 8 Sequence of charge stability diagrams (showing the raw data after subtracting a slow-varying background) demonstrating the single-hole occupancy of Q5b. The presence of Q5b is mainly visible at the charge interdots with the vertical transition lines of Q4b and Q4mb. We indicate the position of the Q5b–Q4b charge interdots. The quasi-horizontal transition line with a positive slope is associated to a spurious dot located in the fanout of plunger line P5 (indicated by a blue dashed line).

Supplementary Video 9 Sequence of charge stability diagrams (displaying the partial derivative along the vP5 axis) demonstrating the third-hole occupancy of both Q5m and Q5t. The vertical transition lines are associated to the Q6 quantum dots.

Supplementary Video 10 Sequence of charge stability diagrams (showing the partial derivative along the vP6 axis) demonstrating the third-hole occupancy of Q6b. The Q6b transition lines have a slight positive slope in the map, whereas the other horizontal transition lines belong to the Q6t quantum dot. To better distinguish the two sets of lines, we step barrier vUB3 that predominantly acts on Q6b, leaving Q6t unperturbed.

Supplementary Video 11 Sequence of charge stability diagrams (showing the partial derivative of raw data along the vP6 axis) demonstrating the single-hole occupancy of Q6t. To better distinguish Q6t from the other transition lines, we plot the signal of the NE charge sensor, and step gate vUB6 that mainly acts on the quantum dot Q6t. By setting the vUB6 barrier more positive to deplete the dot, we also reduce the tunnel coupling to the reservoirs, an aspect that explains the elongated charge interdot with Q7 and the distortion of the Q6t transition line. Supplementary Note 8 and Supplementary Fig. 7 discuss this feature in detail.

Supplementary Video 12 Sequence of charge stability diagrams (showing the partial derivative along the vP7 axis) demonstrating the single-hole occupancy of Q7.

## Data Availability

All data and analysis underlying this study are available via Zenodo at 10.5281/zenodo.8083119 (ref. ^45^).
