# Supplementary material for: Shared control of a 16 semiconductor quantum dot crossbar array
Source: Nat Nanotechnol. 2023 Aug 28;19(1):21–7. doi: 10.1038/s41565-023-01491-3 (PMC10796274; doi:10.1038/s41565-023-01491-3)
Supplement: Supplementary file 1 — Supplementary Figs. 1–17, Tables 1–4 and Notes 1–16. [file 41565_2023_1491_MOESM1_ESM.pdf]

nature nanotechnology

Article https://doi.org/10.1038/s41565-023-01491-3

# Shared control of a 16 semiconductor quantum dot crossbar array

In the format provided by the authors and unedited

CONTENTS

Suppl. Note 1. Rent's rule and gate count S2

Suppl. Note 2. Typical charge sensors response S3

Suppl. Note 3. Virtual gate matrix S4

Suppl. Note 4. Practical device improvements S6

Suppl. Note 5. Quantum dot identification S7

Suppl. Note 6. Spurious quantum dot identification: an example S9

Suppl. Note 7. Demonstration of the odd occupation regime via video sequences S10

Suppl. Note 8. Image correlation techniques for quantum dot detection S11

Suppl. Note 9. Addressable exchange operations with a double barrier design S15

Suppl. Note 10. Two-axis control of the interdot transition line S16

Suppl. Note 11. Two-axis control of the Q6b-Q5m interaction S17

Suppl. Note 12. Electron temperature extraction S18

Suppl. Note 13. Detuning lever arm extraction S19

Suppl. Note 14. Tune-up of the crossbar array in the few-hole regime S20

Suppl. Note 15. Gate voltages of the crossbar in the odd-charge regime S21

Suppl. Note 16. Characterisation of the variability of the quantum dot array S21

References S25

Suppl. Note 1. RENT'S RULE AND GATE COUNT

Scalable architectures impose stringent requirements at all layers of the quantum computing stack [S1]. If we focus on the lowest layers of the computing stack, state-of-the-art solid-state quantum processors do not meet these prerequisites yet [S2]. In fact, current processors still make use of a few control terminals per qubit, an approach that will lead to arduous interconnectivity and control challenges in the route toward millions qubits [S3, S4]. To quantify the level of optimisation and interconnectivity of a quantum processor, we borrow the concept of Rent's rule from classical electronics [S5]. In quantum dot devices, the Rent's rule can be used to correlate the number of control terminals T (i.e., gates, ohmic leads,...) and the number of active components g (i.e., quantum dots or qubits):

$$T = tg^p \tag{1}$$

where t is the average number of control terminals per qubit and p is the Rent exponent that lies in the range $(0, 1]$. Without any quantum hardware optimisation, the number of terminals will keep increasing linearly with the number of qubits, creating major interconnect problems in the quantum computing stack [S2].

An analogous challenge emerged in the 1950s in classical electronics when every electrical component needed to

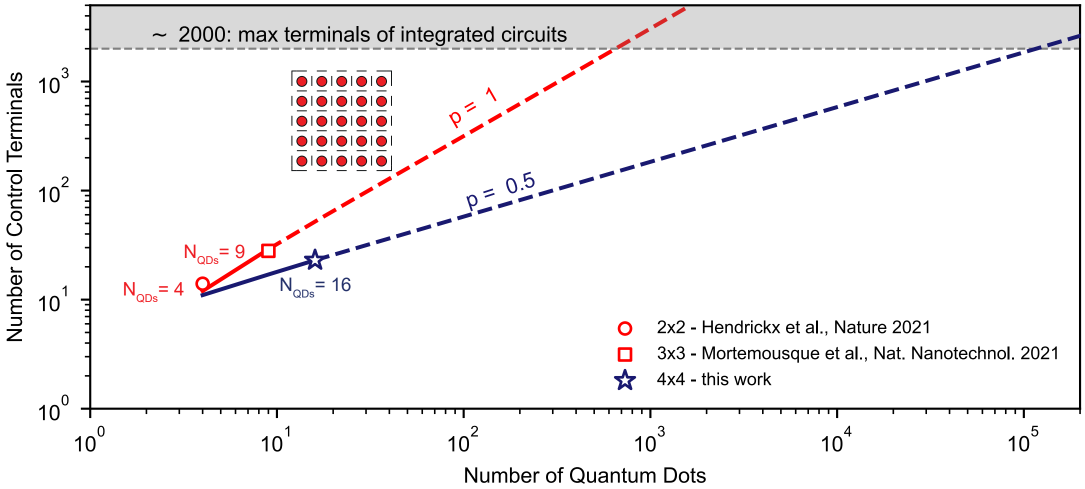

Supplementary Figure 1. Rent's rule in two-dimensional quantum dot architectectures and gates count. Number of control terminals (gates) versus the number of quantum dots for a two-dimensional array. The blue trace indicates the scaling of our shared-control gate architecture. The red trace shows the scaling for an architecture with individual control (inset) of interdot couplings and quantum dot energies. Scatter points represent the gates counts of a 2x2 [S6], 3x3 [S7] and 4x4 quantum dot array (this work). We draw a horizontal line at 2000 control terminals, which currently identify the current maximum number of input/output terminals of classical integrated circuits. Assuming this as a practical limit for the control terminals of a quantum processor without on-chip control logic [S8], the individual control strategy is limited to control up to a few hundred quantum dots. In contrast, the shared control approach may be able to control few hundreds of thousands.

be soldered to several others [S9]. The turning point was the invention of the integrated circuit, which led to the realisation of the first microprocessor - the Intel 4004 - with 2300 transistors and only 16 external pins. Nowadays, integrated circuits are at the heart of our technology and, with a Rent exponent of about 0.5 and a maximum number of input/output lines of $T \sim 2 \cdot 10^3$, present a ratio of transistor to input/output pins g/T of $\sim 10^6$ [S8, S10]. On the contrary, in infant quantum chips, the g/T ratio is below 1 [S2], and therefore, significant efforts have to be made to downscale the quantum Rent exponent.

In our work, we have moved from electrostatic gating strategies with individual control of every unit to crossbar architectures with shared control of quantum dots energies and interdot couplings [S11]. This advance is crucial for scalability because it may enable to use $O(N^{1/2})$ terminals for $O(N)$ qubits (i.e., $p = 0.5$). The impact of this strategy can be visualised in Suppl. Fig. **1**, where we compare the different scalings of control terminals for a two-dimensional quantum dot array controlled with a shared- and with an individual-control approach. For the shared-control architecture presented in this work, the gates count as a function of quantum dots g is given by:

$$T = 6g^{1/2} - 1 \propto 6g^{1/2} \tag{2}$$

while for an architecture with individual control of all the on-site dot energies and interdot couplings (see inset of Suppl. Fig. **1**), we obtain:

$$T = 3g + 2g^{1/2} - 4 \propto 3g \tag{3}$$

These equations hold for square arrays with a minimum 2x2 size, i.e., $g \geq 4$.
Scaling the crossbar architecture to a 6x6 crossbar array would already result in a device with fewer gates than total number of quantum dots. In particular, a 6x6 crossbar array with 36 quantum dots requires only 35 gates. We note that while this may seem a marginal improvement, comparing the required number of gates to quantum dot devices with individual connectivity shows already a remarkable difference: a 4x4 (6x6) quantum dot device requires 23 vs 52 (35 vs 116), for the respective systems.
We emphasise that in these considerations we do not account for the terminals controlling the read-out charge sensors. In the future, a strategy for integrating charge sensors within quantum dot crossbar arrays needs to be fully worked out to establish and operate scalable modules of semiconductor qubit arrays with a Rent exponent of 0.5. We note that germanium can make ohmic contacts to metals, thus facilitating a very small footprint for charge sensors, and providing a route toward the integration of charge sensors in the quantum dot array.

Suppl. Note 2. TYPICAL CHARGE SENSORS RESPONSE

To read out the charge states of the 16 quantum dots, we prepare four charge sensors in the Coulomb regime, as demonstrated by the transport features in Suppl. Fig. **2**, and operate them at the steepest points.

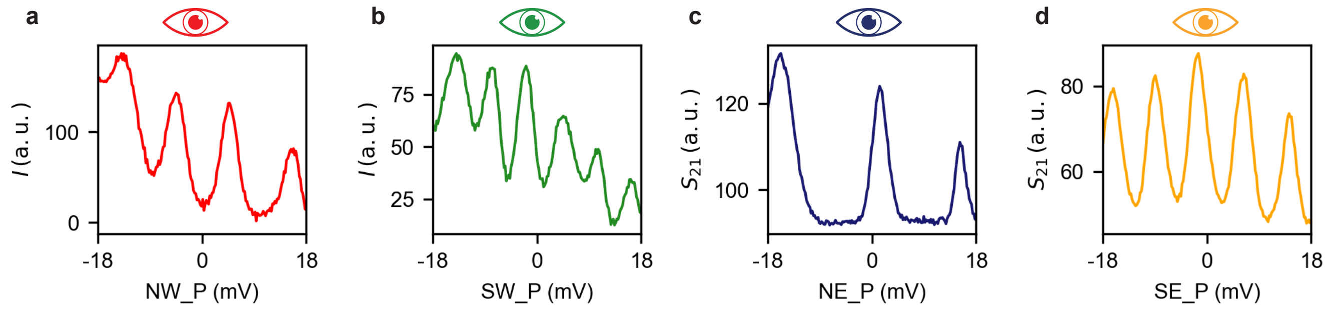

Supplementary Figure 2. **Typical charge sensor responses. a, b** Direct-current I via the NW and SW sensor. **c, d** Reflectometry signal S_{21} of the NE and SE sensor respectively. The traces are taken as a function of the relative sensor plunger gate.

Suppl. Note 3. VIRTUAL GATE MATRIX

In Suppl. Fig. **3** we display the virtual matrix used in software to define the set of virtual gates. We define the virtual gates such that their variation does not influence the position of the charge sensors Coulomb peak. Concretely, we perform in video-mode fast 2D gate scans of the kind NW_P vs LB1. We then identify the slope of the position of the sensor Coulomb peak in such a 2D map, and set this value to the matrix element (NW_P, vLB1). This method results in the definition of a virtual gate vLB1 that maintains the NW charge sensor tuned. This procedure is iterated across all plunger and barrier gates, and all the charge sensors.
Similarly, virtual plungers are also designed to be able to tune independently the on-site energies of the quantum dots by using only nearest-neighbours compensations as described in refs. [S12, S13]. Because multiple sites are controlled by a single plunger line and each of the site can have a slightly different crosstalk to the surrounding gate, our procedure results in an approximation. We note that in this work we have not virtualised the barrier gates beyond the two double dots considered in Fig. 4.

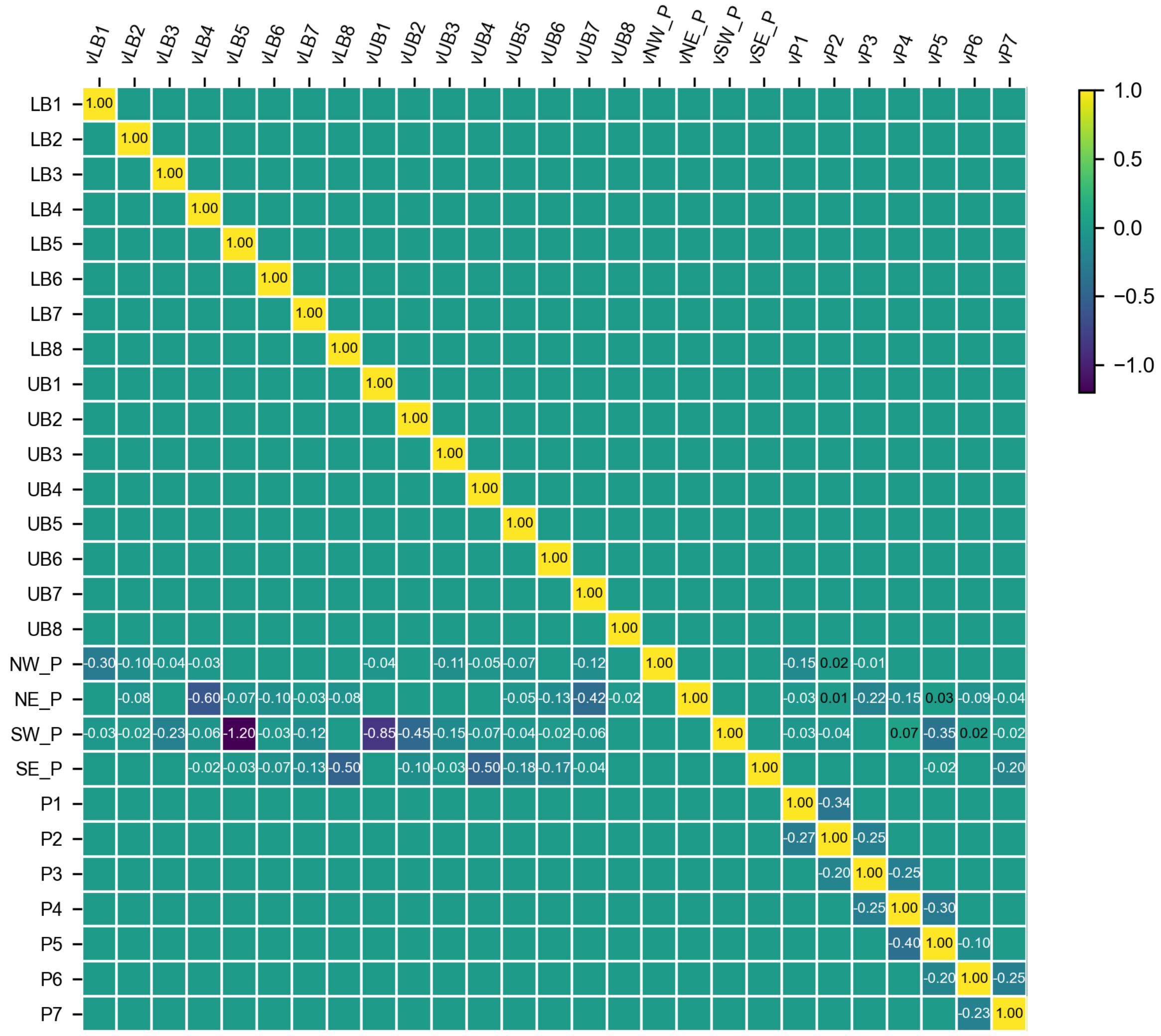

Supplementary Figure 3. **Virtual gate matrix.** Visualization of the virtual gate matrix. Virtual barriers vLBi and vUBi are defined as combination of the relative barriers LBi and UBi with $i \in [1, 8]$ and sensor plungers. NW_P, NE_P, SW_P and SE_P identify the plungers of the respective charge sensors.

Suppl. Note 4. PRACTICAL DEVICE IMPROVEMENTS

Here, we present a series of practical ideas to mitigate challenges observed (and not) throughout the experiment.

1. The tune-up of the current implementation is complicated by the emergence of a few (~ 5) unwanted stray dots outside the array, located under the dense gates fanout. These decrease the read-out sensitivity to the designed quantum dots and, in general, complicate the system tune-up.
   Near-term solutions can be: the addition of a lower "screening" gate layer (kept at a more positive voltage), or an upper "depleting" gate layer that prevents any charge puddles from forming in between different gate lines. A more sophisticated solution consists of the implementation of vertical interconnect access through the oxide layer that enables to fan out the gate lines at a much higher level in the stack [S14]. We also envision that, in the future, boundary effects of large qubit arrays need to be accounted for (e.g., dots at the perimeter of the array experience a smaller electric field than the ones in the middle). Hence, another possibility is to neglect the dots at the perimeter completely.

2. A faster device tunability can be achieved by using radio frequency reflectometry, which, in our experiment, worked out only partially.

3. A higher level of homogeneity and functionality than is presented here will be needed for practical quantum computation with quantum dots. Leaving aside the development on the material stack itself (Ge/SiGe) and on the device nanofabrication, there appears to still be space for improvements on the gate layout.
   Similarly to the behaviour of the turn-on voltages in transistors [S15], a higher quantum dot homogeneity may be achieved by increasing their size. This will also have the beneficial effect of increasing the plunger gates lever arm, which will be less screened by the lower layers. Clearly, an excessively large quantum dot can lead to (Pauli spin-blockade) read-out problems if the energy of the first excited state is too low. Therefore a search for an optimal size needs to be performed.

4. Other strategies can be adopted in the near future to further improve the level of quantum dot uniformity. In particular, the method recently presented in ref. [S16] relies on engineering the electrostatic landscape using the hysteretic shift of the gate voltage characteristics.

5. In the current device, we observed that the ratio of the two barrier layers lever arms is approximately 2-3. Due to imperfections in the nanofabrication and an oxide layer in between them, the top layer partially overlaps the bottom one. As a consequence, its electric field is screened. To enhance the coupling of the top layer, one can design the top layer to have a slightly larger width, or add a gap of a few nm in between the two lines.

Suppl. Note 5. QUANTUM DOT IDENTIFICATION

To obtain the capacitive coupling of each barrier to each dot, we analyse several charge stability diagrams as shown in Suppl. Fig. **4**a. We monitor the position of the transition lines (away from charge interdots) by fitting the derivative of the data with a Gaussian function, after subtraction of a slow-varying background. In the small voltage range that we consider, the extracted peak positions respond linearly to each barrier gate (Suppl. Figs. **4**b, c) The normalised slope of the fitted linear function is used to quantify the capacitive coupling of each gate. The latter are plotted as histograms and visualised on the device layout in Suppl. Fig. **5**. As described in the Methods, this information can be used to clearly assign each set of transition lines to the dot in the grid.

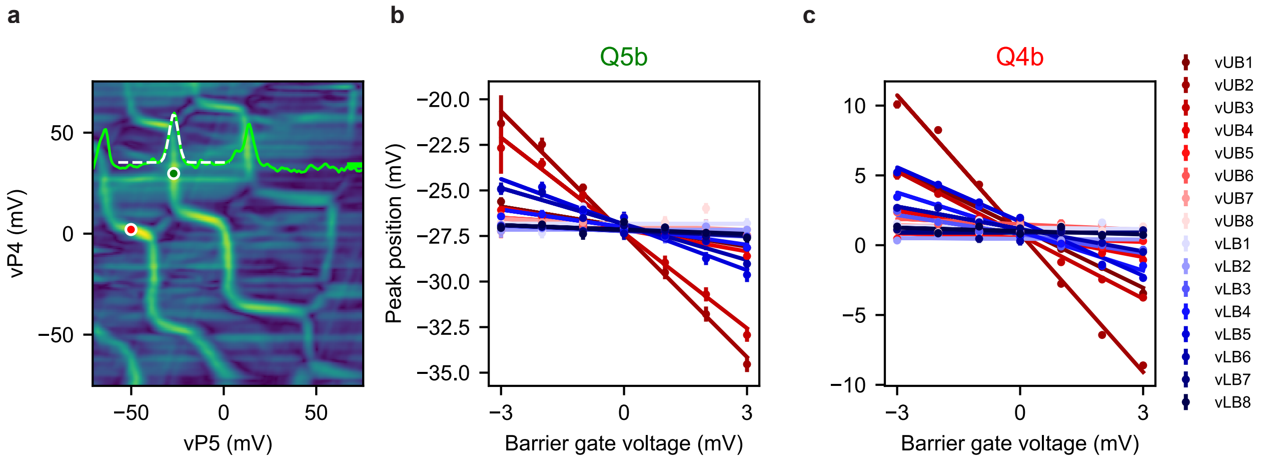

Supplementary Figure 4. Detecting the shift of the transition lines: an exemplary case. a, Charge stability diagram (gradient of the SW sensor signal) showcasing a set of vertical and horizontal charge transition lines. The green trace shows the derivative of the data at the vP4 value identified by the horizontal green tick. The white dashed line is a fit of the linecut with a Gaussian function, and the green marker identifies the fitted centre of the peak. The red marker labels the fitted coordinate of a vertical transition line (not shown). **b, c,** Scatter points are the fitted positions of the green and red markers, respectively, as a function of the voltage applied at all barriers. Error bars on the points display the standard deviation of the fitted centre of the Gaussian. Dashed lines are the best linear fit to the data. The normalised absolute values of the slope parameter are taken as capacitive couplings of each gate to the specific transition line. The error bar on the capacitive coupling is taken as one standard deviation of the fitted slope parameter. The horizontal (vertical) transition line in **(a)** is attributed to the quantum dot Q4b (Q5b).

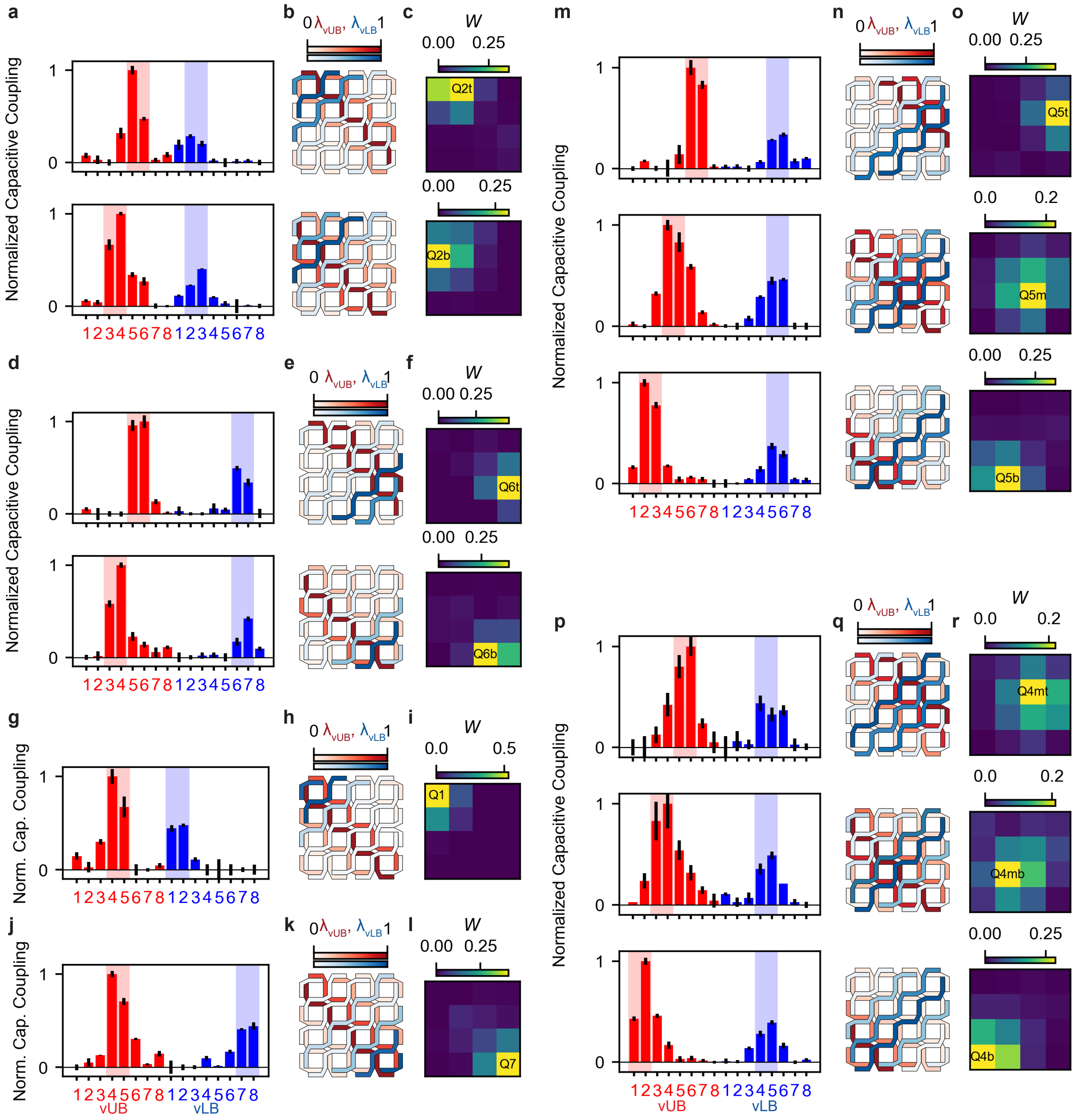

Supplementary Figure 5. **Quantum dot identification.** **a,** Histograms of the (normalised) capacitive couplings (in red for the vUB, and in blue for the vLB gates) obtained by the analysis of transition lines attributed to different quantum dots as shown in Suppl. Fig. **4**. Red and blue backgrounds are added to emphasise the two barriers that surround the labelled quantum dot. **b,** Device layout with the capacitive couplings colour-coded on the filling of the gate lines. The vUB (vLB) capacitive couplings are normalised to their maximum values. Intuitively, the quantum dot associated with the analysed transition lines is located at the intersection of the two intensely coloured red and blue lines. **c,** Extracted probabilities (W) of each set of addition lines (calculated as discussed in the Methods). The comparison of the top and bottom panels of **a, b, c** (**d, e, f**) clearly distinguishes the two Q2 (Q6) quantum dots. Similarly, our method is applied on the two dots controlled by independent plunger lines Q1 and Q7 in panels **g, h, i** and **j, k, l**, respectively. The three different rows of panels **m, n, o** (**p, q, r**) enables to label the Q5 (Q4) quantum dots. In **a, d, g, j, m, p** the data points correspond to the peak of the chart bars as well as the centers of the error bars. Each error bar is the standard deviation of the parameter obtained from the linear fit. The dot Q4t could not be systematically analysed because, in this gating regime, we observe a slow loading mechanism via the defective barrier UB8 with respect to the timescale of our scan (∼ ms). However, because such transition lines are controlled by vP4 (labelled in Suppl. Fig. **14**), do strongly respond to vUB7 and vLB5, we can still map them to the site Q4t, in a qualitative way.

Suppl. Note 6. SPURIOUS QUANTUM DOT IDENTIFICATION: AN EXAMPLE

We make use of the method presented above to also map the position of accidental quantum dots that arise outside the crossbar array. In Suppl. Fig. **6**a, we display the capacitive coupling of the barrier gates to a spurious quantum dot. The addition lines of such a quantum dot are mainly controlled by vP2 and are visible as quasi-vertical lines in the charge stability diagrams of the type ΔvP2 vs ΔvP3 in the Supplementary Videos. In particular, from these maps, it is possible to observe a negligible mutual capacitance between this specific accidental dot and the crossbar quantum dots Q1, and Q3b. However, we emphasise that the presence of such spurious quantum dot complicates the tuning efficiency of our device, and strategies to mitigate their presence need to be in place in the near future, as already discussed in the main text. In Suppl. Fig. **6**b, we present the device layout with the capacitive couplings colour-coded on the filling of the gate lines. Here, both the vUB and vLB capacitive couplings, λ_{vUB} and λ_{vLB}, are normalised to their maximum values. Following this analysis, we can conclude that such spurious quantum dot is approximately located under the fanout of the gates UB1, LB3 and UB3. This is furthermore corroborated by the fact that this dot is well sensed by the SW charge sensor.

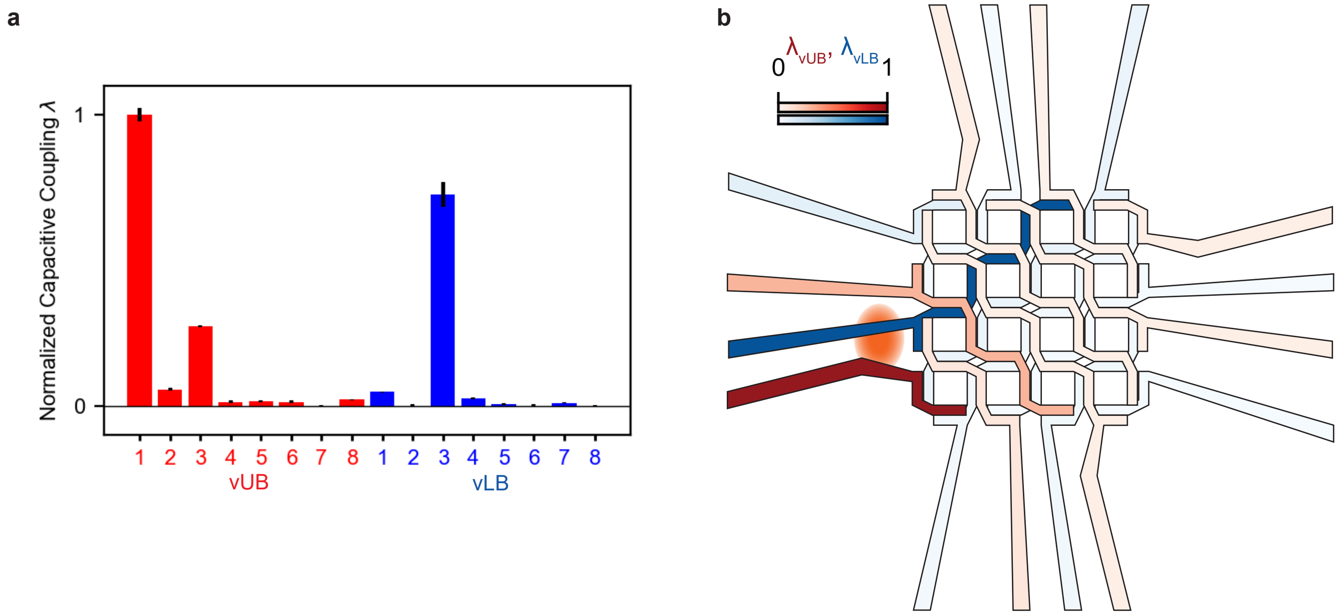

Supplementary Figure 6. **Spurious quantum dot identification. a,** Histograms of the (normalised) capacitive couplings (in red for the vUB, and in blue for the vLB gates) obtained from the analysis of transition lines attributed to a spurious quantum dot located under the gates fanout. The data points correspond to the peak of the chart bars as well as the centers of the error bars. Each error bar is the standard deviation of the parameter obtained from the linear fit. **b,** Device layout with the capacitive couplings colour-coded on the filling of the gate lines. The vUB (vLB) capacitive couplings are normalised to their maximum values. The orange oval in **(b)** indicates the approximate position of the quantum dot associated with the analysed transition lines (under the fanout of the barriers UB1 and LB3).

Suppl. Note 7. DEMONSTRATION OF THE ODD OCCUPATION REGIME VIA VIDEO SEQUENCES

We have combined the stepping charge stability diagrams into Suppl. Videos 1-12. In these videos, we present sequences of charge stability diagrams of the type vPx versus vPy with $x, y \in [1, 7]$ and $x \neq y$. Because the maximum amplitude of the arbitrary waveform generator (AWG) ramps at the device is at max $\sim$ 200 mV due to the attenuation in the lines, a single two-dimensional scan is not enough to evaluate directly the number of holes in a quantum dot. Rather, we start from the gate voltage regime presented in the main text Fig. 3 and proceed by increasing the dc voltage vPx in steps of 10 mV toward more positive voltages until Qx is fully depleted. At every step, we take a fast two-dimensional scan that allows to label and count the number of transition lines visible in the available gate window. The size of the visible zeroth charge sector is always chosen in all the cases to be bigger than the relative first hole spacing ($\sim$ 50 mV), see Suppl. Table **1**. We present these measurements starting from an empty dot and finishing in the original configuration, with a square labelling the (0,0) point in the map signalling the odd occupancy regime. In analogy to the main text, the red, blue, green and yellow frames of the plots indicate that the data have been measured with the NW, NE, SW and SE charge sensor, respectively.

In Supp. Table **2**, we list the dc virtual plunger voltages of the datasets presented as Supplementary Videos.

**Quantum dot**	**Size of the visible 0th charge state (mV)**	**1st addition voltage (mV)**	**Ratio**
**Q1**	111	49	2.3
**Q2b**	120	46	2.6
**Q2t**	116	46	2.5
**Q3b**	71	54	1.3
**Q3m**	78	46	1.7
**Q3t**	77	40	1.9
**Q4b**	96	52	1.8
**Q4mb**	101	52	1.9
**Q4mt**	105	64	1.6
**Q4t**	125	47	2.7
**Q5b**	107	48	2.2
**Q5m**	125	54	2.3
**Q5t**	125	65	1.9
**Q6b**	66	50	1.3
**Q6t**	105	50	2.1
**Q7**	75	44	1.7

Supplementary Table 1. Comparison of the size of the visible 0th charge state in the charge stability diagrams with the addition voltage of each quantum dot. The range in which no transition lines are observed is always larger than 1.3 times the spacing of the first two transition lines.

**Supplementary Videos**	**vP1(mV)**	**vP2 (mV)**	**vP3(mV)**	**vP4 (mV)**	**vP5 (mV)**	**vP6 (mV)**	**vP7 (mV)**
**1**	-1369	-1354	-1527	-2056	-1995	-1612	-1380
**2**	-1369	-1354	-1527	-2056	-1995	-1612	-1380
**3**	-1363	-1354	-1527	-2056	-1995	-1612	-1380
**4**	-1363	-1354	-1527	-2056	-1995	-1612	-1380
**5**	-1363	-1354	-1527	-2056	-1995	-1612	-1380
**6**	-1363	-1354	-1527	-2056	-1995	-1612	-1380
**7**	-1363	-1354	-1527	-2056	-1995	-1612	-1380
**8**	-1363	-1354	-1527	-2056	-1995	-1612	-1380
**9**	-1363	-1354	-1527	-2056	-1995	-1612	-1380
**10**	-1369	-1354	-1527	-2056	-1995	-1612	-1379
**11**	-1369	-1354	-1527	-2056	-1995	-1612	-1380
**12**	-1369	-1354	-1527	-2056	-1995	-1612	-1378

Supplementary Table 2. **Virtual plunger voltages.** Table listing all the dc voltages applied to the virtual plunger gates underlying the measurements presented in the Supplementary Videos 1-12. The largest variation (6 mV) on vP1 does not affect the occupancy of Q1 (neither the surrounding Q2b and Q2t quantum dots), as can be seen in Suppl. Videos 1 and 2.

Suppl. Note 8. IMAGE CORRELATION TECHNIQUES FOR QUANTUM DOT DETECTION

Verifying the odd charge state requires tracking the charge addition lines of all 16 quantum dots. This can be a tedious task as the Supplementary videos in the previous subsection demonstrate because it involves studying several charge stability maps at different plunger gate configurations with varying visibility. Some charge transition lines only can be reconstructed by their interdot transitions with other dots, and slow tunnel couplings to the reservoirs (Suppl.Fig. **7**) cause postponed loading (i.e. latching) which further complicates the interpretation [S17–S20]. Therefore, we have developed an algorithm that unifies and simplifies the assessment of the first few charge transition lines of a given quantum dot to ease the determination of its charge occupation.

In order to track the transitions of a given quantum dot Qx, we acquire charge stability maps M_n^{raw} sweeping the

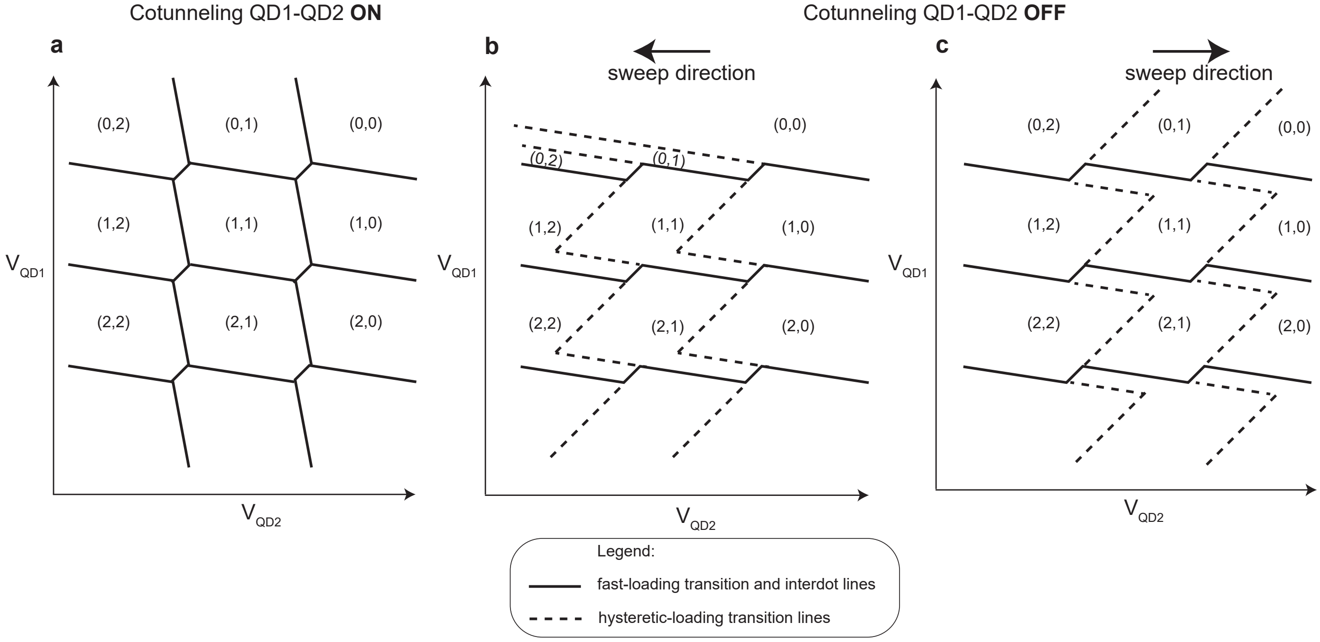

Supplementary Figure 7. **Effects of low tunnel rate to the reservoir in charge stability diagrams. a,** Cartoon of a conventional honeycomb charge stability diagram of a double dot system where the reservoir tunnel rates to both dots Q1 and Q2 are much higher than the sweep rate of both axes. If one dot (Q2) is located further away from the reservoir than the other (Q1), loading in Q2 occurs thanks to cotunnelling events via Q1. **b, c** When Q2 is coupled to the reservoir at a rate much slower than the sweeping time, the charge stability map displays hysteretic features whose occurrences depend on the sweep direction [S17]. Q2 is then loaded via the extended charge interdots of Q1-Q2, which remains active due to the finite Q1-Q2 interdot coupling. We note that when the charge stability diagrams display such features, the interdots with Q1 still enables to reconstruct the Q2 transition line at equilibrium. We also emphasise that typical maps manifest a behaviour that is in between the case displayed in **(a)** and in **(b)** (or **(c)**) depending on the sweep direction and the tunnel rate to the leads, which is typically non zero.

respective plunger gate vPx against a neighbouring plunger gate vPy for N_{scan} constantly spaced offset voltages $V_{\mathrm{off},n}$ applied to vPx or a third gate (as presented in the Supplementary Videos). The charge stability maps are preprocessed by applying an individual combination of background subtraction, derivatives, local contrast normalisation, cropping along the secondary axis (vPy) and normalisation as detailed in Table **4**. A reference feature F is then defined as a renormalised section of one of the preprocessed charge stability maps M_n. It is chosen to uniquely identify the respective quantum dot transition line, either directly containing a section of a transition line or showing an interdot transition with an adjacent dot.

Next, we calculate the image correlation C_n of the charge stability maps M_n with the reference feature F:

$$C_n(\mathrm{vP}x, \mathrm{vP}y) = \sum_{\Delta \mathrm{vP}x, \Delta \mathrm{vP}y} M_n(\mathrm{vP}x + \Delta \mathrm{vP}x, \mathrm{vP}y + \Delta \mathrm{vP}y) F(\Delta \mathrm{vP}x, \Delta \mathrm{vP}y)$$

Typically C_n will exhibit a maximum at the gate voltages for which F was defined. However, often F also has an increased correlation with similar features of higher or lower transition lines enabling the detection of those as well.

We note that in some cases we redefine the feature F to compensate for sensor shifts or features changing with the charge occupancy of the dot (e.g. growing interdot transitions). The number of feature definitions per number of charge stability diagrams is given in the last column of Table **4**. Each correlation map C_n is then divided into sub-parts $C_{n,m}$ along the vPx axis and the maximum correlations $\hat{C}_{n,m} = \max(C_{n,m})$ and the respective coordinates $(\mathrm{v\hat{P}}x_{n,m}, \mathrm{v\hat{P}}y_{n,m})$ are determined. In particular we choose four sub-parts ($m \in \{0, 1, 2, 3\}$) for all quantum dots corresponding to a spacing similar to typical charging voltages and thus increasing the probability of only a single transition line crossing each sub-part. To minimise the number of falsely positive detections which are not related to actual occurrences of transition lines we furthermore apply a threshold keeping only $(\mathrm{v\hat{P}}x_{n,m}, \mathrm{v\hat{P}}y_{n,m})$ with $\hat{C}_{n,m} \geq 0.35 \times \max_{m,n}(\hat{C}_{n,m})$. In Fig. 3 in the main text we plot the remaining $(\mathrm{v\hat{P}}x_{n,m}, \mathrm{v\hat{P}}y_{n,m})$ as a function of n with $\hat{C}_{n,m}$ encoded in the individual colouring of the scatter points.

The correlation maxima $\hat{C}_{n,m}$ corresponding to a specific charge transition $i \to j$ are expected to shift from charge map to charge map by a regular voltage shift $\frac{\Delta \mathrm{v\hat{P}}x_n}{\Delta n}$, thus falling on a line $\mathrm{v\hat{P}}x^{i\to j}$ given by:

$$\mathrm{v\hat{P}}x^{i\to j}(n) = \frac{\Delta \mathrm{v\hat{P}}x_n}{\Delta n} \times n + \mathrm{v\hat{P}}x_0^{i\to j}$$

Note that we dropped the m subscripts as with increasing n the transition line can shift from one sub-part to another. We utilise this predictable behaviour to increase robustness against occasionally missed features and further reduce the effect of falsely obtained correlation maxima which do not correspond to a a charge transition and thus typically shift randomly from charge map to charge map. To that end we define a dense set of potential transition lines

$$\mathrm{v\hat{P}}x^{B}(n) = \frac{\Delta \mathrm{v\hat{P}}x_n}{\Delta n} \times n + B$$

with B stepped in steps of 4 mV from $B_{\mathrm{min}} \ll \min(\mathrm{vP}x)$ to $B_{\mathrm{max}} \gg \max(\mathrm{vP}x)$. The chosen $\frac{\Delta \mathrm{v\hat{P}}x_n}{\Delta n}$ are given in Table **3**. Most values are close to 10 mV as for most quantum dots we step the vPx gate offset with each charge stability map by 10 mV. Small deviations from 10 mV arise due to imperfect calibrations of the AWG voltages with respect to the Digital to Analog Converted (DAC) voltages. For each B we then identify all the scatter points $(\mathrm{v\hat{P}}x_{n,m}, \mathrm{v\hat{P}}y_{n,m})$ that are located in a range of $\pm$ 4 mV around $\mathrm{v\hat{P}}x^{B}(n)$. These then allow for all lines $\mathrm{v\hat{P}}x^{B}(n)$ to determine a heuristic measure of likelihood to be an actual transition line. We calculate this likelihood $p(B)$ using the following equation, that keeps into account the number of points N_B falling in that range, their correlation $\hat{C}_{n,m}$ and the maximum number of feature occurrences that could have been detected N_B^{max}:

$$p(B) = \frac{1}{N_B^{\mathrm{max}}} \left[-L \times (N_B^{\mathrm{max}} - N_B) + \sum_{\substack{\hat{C}_{n,m} \text{ if} \\ \mathrm{v\hat{P}}\, x^{B}(n) - 4 \text{ mV} \\ \leq \mathrm{v\hat{P}}x_{n,m} \leq \\ \mathrm{v\hat{P}}x^{B}(n) + 4 \text{ mV}}} \max_m(\hat{C}_{n,m}) \right]$$

Here $L = 0.3 \times \max_{m,n}(C_{n,m})$ defines a penalty term lowering $p(B)$ for every potential feature occurrence that has not led to a detected correlation maximum. Fig. 3 in the main text shows the set of lines $\mathrm{v\hat{P}}x^{B}(n)$ with $p(B)$ colour-coded in the colour of each trace. A few lines clearly emerge from the others, identifying the charge transitions of the various quantum dots. The white square with black outline marks the final voltage setpoint, which is clearly located between two dense clusters of high-$p(B)$ lines. In Suppl. Fig. **8**, we combine the results of the method applied for all quantum dots, by showing a line-cut of $p(B)$ on the rightmost part of the panels ($n = N_{\mathrm{scan}}$) presented in main text Fig. 3. Charge transitions are visible as peaks, and are fitted with a Gaussian function to extract their position. From this analysis, we obtain a $\sim$ 7 mV virtual plunger voltage range for the odd charge regime presented here. We emphasise that gate voltages have not been optimised to centre the charge symmetry points of the quantum dots at the tuning point, but this may be required for qubit operation.

The detection precision of the resulting transition voltages for each dot depends on the specific underlying set of charge stability diagrams. For transition lines perfectly aligned with the vPy-axis the potential variation of the vPx-coordinate is minimised. However, in practice residual cross capacitances not compensated by the virtual gates remain leading to sloped transition lines. As a given feature often can reappear multiple times on the same transition line at differing vPy voltages this then results in a scattering of the vPx-coordinates around the expected behaviour.

Also, latching effects depending on the scan ranges of vPx and vPy can cause those variations. However, careful feature definitions and reduced vPy scan ranges lower this impreciseness. Furthermore, we also correct for constant offsets between the feature positions and the charge transition line voltages.
While here our method is utilised to unify and ease the tedious task of tracking the full spectrum of charge transitions for all dots which still requires careful prepossessing and a manual selection of features we envision that in future work it could become part of automatic tuning and verification procedures.

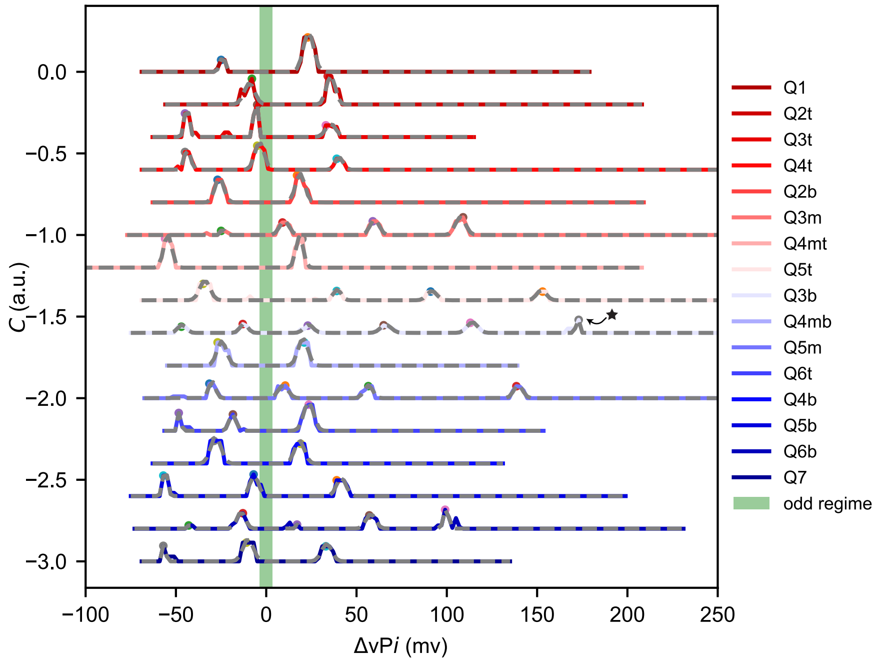

Supplementary Figure 8. **Charge transition lines of all the quantum dots in the odd charge occupancy regime** We plot the likelihood quantity $p(B)$ for all the quantum dots taken at the position of the white square marker in the panels of main text Fig. 3 (i.e., at the max $n = N_{\text{scan}}$) and thus for the offset voltage configuration of the odd charge state. Charge transition lines identified via image detection methods appear as peaks. We fit the sequence of peaks with Gaussian functions to extract their position in the voltage space. The star on the first peak of the quantum dot Q3b indicates a false-positive transition line, as discussed in the main text. The green rectangle identifies the span in voltage of all the virtual plungers in which the odd occupancy is conserved. The broadening of the peaks is mainly due to the jitter in high-correlation points $(\text{v}\hat{\text{P}}x_{n,m}, \text{v}\hat{\text{P}}y_{n,m})$ as visible in main text Fig. 3. We note that the lines cover the range set by the algorithm, which is larger than the range spanned by the data.

**Stepped gate**	$\frac{\Delta \text{v}\hat{\text{P}}x_n}{\Delta n}$ **(mV/scan)**
vP1	10.75
vP2	10.0
vP3	10.0
vP4	9.0
vP5	12.0
vP6	8.7
vP7	10.0
vUB3	7.5
vUB6	6.0
vUB7	5.4

Supplementary Table 3. Slope coefficient $\frac{\Delta \text{v}\hat{\text{P}}x_n}{\Delta n}$ of the transition lines in the panels of the main text Fig. 3.

**Quantum dot**	**background subtraction**	**derivative**	**local contrast normalisation**	**cropping along vPy**	**number of defined features per number of charge maps**
Q1	no	$dM_n/d\mathrm{vP}x + dM_n/d\mathrm{vP}y$	yes	yes	1/6
Q2t	no	$dM_n/d\mathrm{vP}x$	no	yes	1/8
Q2b	no	$dM_n/d\mathrm{vP}x$	no	yes	1/8
Q3t	no	$dM_n/d\mathrm{vP}y$	yes	yes	2/8
Q3m	yes	no	no	yes	1/12
Q3b	no	$dM_n/d\mathrm{vP}x$	no	no	1/12
Q4t	no	$dM_n/d\mathrm{vP}x$	yes	yes	1/12
Q4mt	yes	no	no	yes	1/12
Q4mb	no	$dM_n/d\mathrm{vP}x$	no	yes	1/4
Q4b	no	$dM_n/d\mathrm{vP}x$	no	yes	1/4
Q5t	no	$dM_n/d\mathrm{vP}x$	yes	yes	2/16
Q5m	no	$dM_n/d\mathrm{vP}x$	no	yes	2/16
Q5b	no	$dM_n/d\mathrm{vP}x$	yes	yes	3/6
Q6t	no	$dM_n/d\mathrm{vP}x$	no	no	1/8
Q6b	no	$dM_n/d\mathrm{vP}x$	yes	yes	2/12
Q7	no	$dM_n/d\mathrm{vP}x$	no	no	1/4

Supplementary Table 4. Preprocessing applied to the raw charge stability maps M_n^{raw} to obtain M_n and number of defined charge transition features. Here, background subtraction refers to subtracting a smoothed version of the charge stability map: $M_n^{\mathrm{out}} = M_n^{\mathrm{in}} - M_n^{\mathrm{in}} * f_{\mathrm{Gaussian}}$. Local contrast normalisation corresponds to $M_n^{\mathrm{out}} = \frac{M_n^{\mathrm{in}} - M_n^{\mathrm{in}} * f_{\mathrm{Gaussian}}}{\sqrt{(M_n^{\mathrm{in}} - M_n^{\mathrm{in}} * f_{\mathrm{Gaussian}})^2 * f_{\mathrm{Gaussian}}}}$. In both cases M_n^{in} and M_n^{out} refer to the charge stability diagram before and after the respective processing and f_{Gaussian} is a Gaussian distribution with a standard deviation of 7-10 pixels and 4-10 pixels respectively. The postprocessing steps are applied in the order as presented in the table (from left to right). In all cases the final charge stability map M_n is normalised such that $0 \leq M_n(\mathrm{vP}x, \mathrm{vP}y) \leq 1$. The last column provides the number of defined transition features F and the total number of charge stability maps M_n (N_{scan}).

Suppl. Note 9. ADDRESSABLE EXCHANGE OPERATIONS WITH A DOUBLE BARRIER DESIGN

The ability to control the tunnel coupling with two barriers opens the opportunity to design addressable exchange-based two-qubit gates in architectures with shared control. We envision an operation strategy in which a two-qubit gate can be activated only when both barriers are in the ON state. For a fast CPHASE gate with a duration of 5 ns, we require to activate an exchange interaction of $J_{\mathrm{ON}}/h = 100$ MHz. In all other cases (i.e., when the barriers are in the configurations (ON, OFF), (OFF, ON), (OFF, OFF)), we demand a sufficiently low exchange to minimise errors. State-of-the-art values of OFF exchange interaction are in the order of $J_{\mathrm{OFF}}/h \sim 10$ KHz [S21].

Suppl. Fig. **9** illustrates the required barrier voltage points to obtain such exchange interactions considering symmetric lever arms, a quantum dot charging energy of U = 1 meV and operations at the charge symmetry point (i.e., at zero detuning). In Suppl. Fig. **9**, we have approximated the dependence of the tunnel coupling energy t_{C} with respect to the two barrier voltages B_x and B_y with [S22–S24]:

$$t_{\mathrm{C}} = \frac{\sqrt{JU}}{2} = c_1 \cdot e^{-c_2\alpha(B_x - B_{x,ON})} \cdot e^{-c_2\alpha(B_y - B_{y,ON})} \tag{4}$$

with $B_{x,ON}$ and $B_{y,ON}$ the ON set-points of the two barriers.
In this example, we have set the prefactor c_1 to 5·h GHz with h the Plank constant, and the effective barrier lever arms $c_2 \cdot \alpha$ to 0.04 V^{-1}.

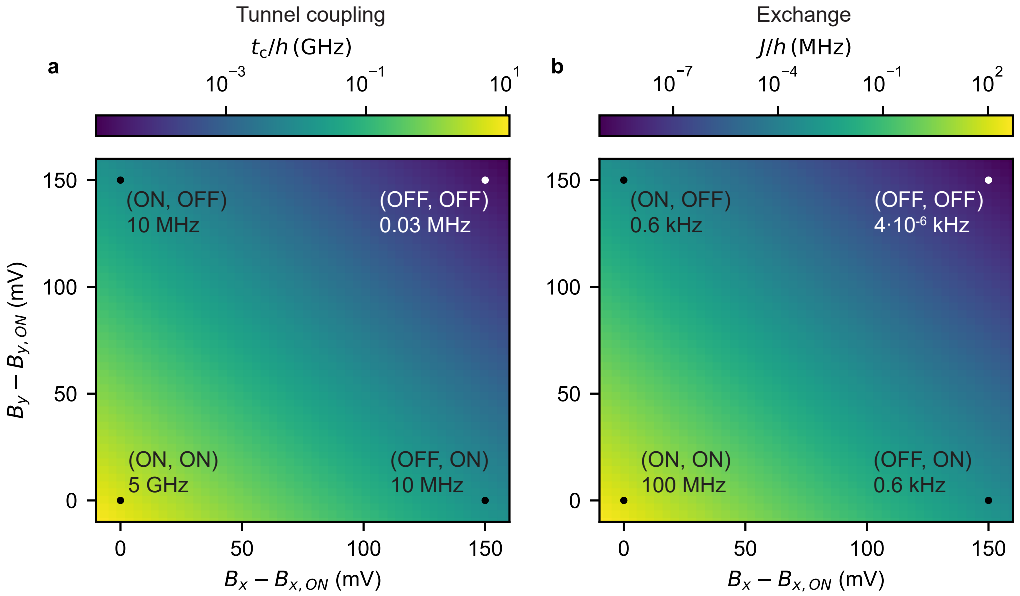

Supplementary Figure 9. **Addressable exchange operation with a double barrier design. a, b** Target tunnel coupling and exchange required for fast two-qubit gates with interaction ON (bottom left corner), and OFF (top left, top right, bottom left corners in the map). The exchange interaction of qubit pairs at (ON, OFF) crossing points remains four orders of magnitude smaller than in the (ON, ON) cases. The parameters displayed are calculated at the coordinates (0, 0), (0, 150), (150, 0), (150, 150) mV. In reference to the experimental results of Fig. 4, the measurable tunnel coupling range (> 1 GHz) via polarisation lines is the bottom left corner of panel **(a)** extending up to +24 mV on both axes.

Suppl. Note 10. TWO-AXIS CONTROL OF THE INTERDOT TRANSITION LINE

By fitting the two-dimensional (e67, U67) charge stability diagrams (examples in Suppl. Figs. **10**a, b), we obtain an estimate of the (3,1)-(2,2) charge interdot size L for the double dot systems Q6b-Q7 and Q6t-Q7. The size of the interdot line is indicative of the capacitive coupling between the adjacent quantum dots. Consistent with the two-axis tunability of the tunnel coupling, the interdot size is varied as the effective distance between the dots is modified by the action of the two tunnel barriers (Suppl. Figs. **10**c-f).

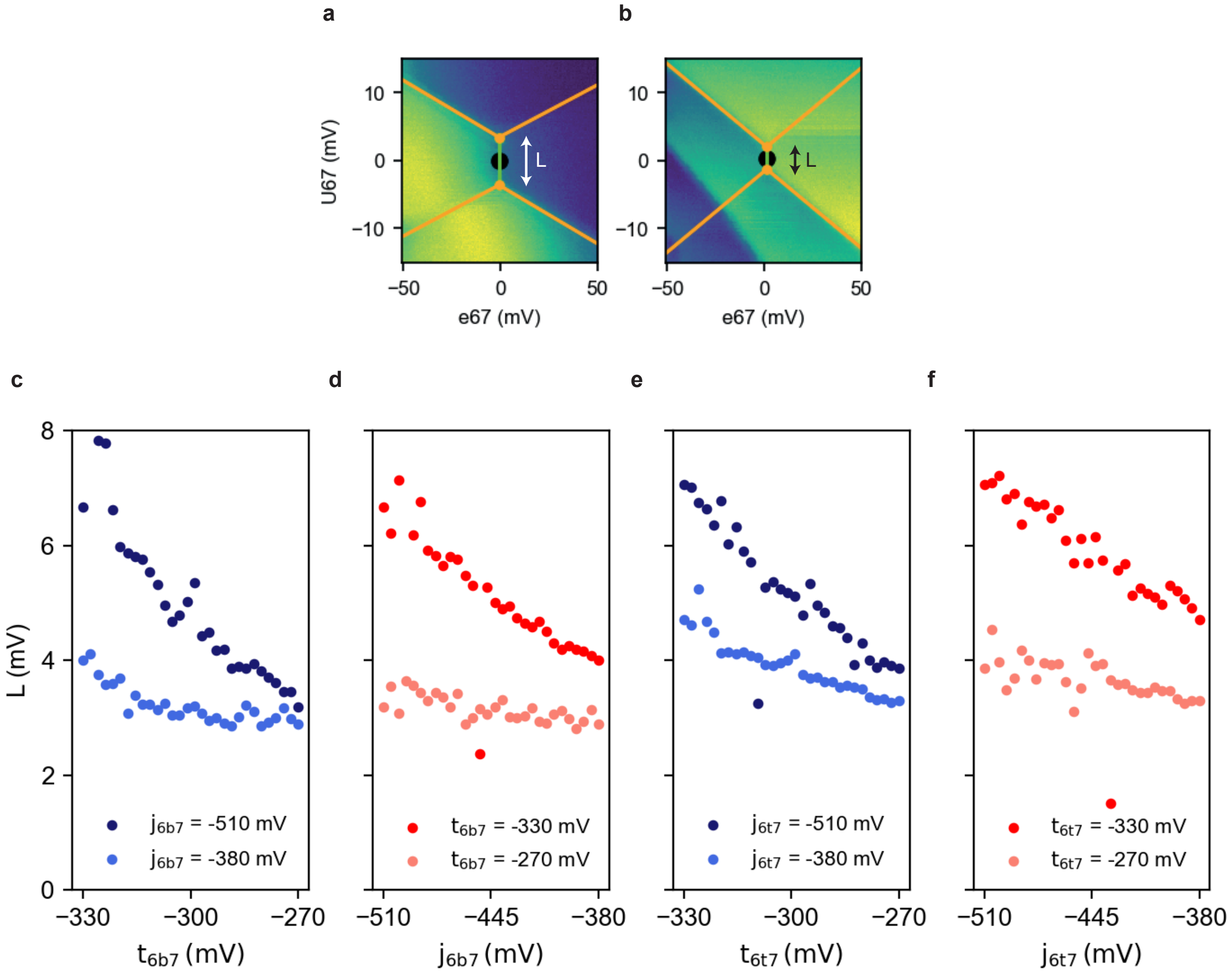

Supplementary Figure 10. **Two-axis control of the quantum dot interdot transition line. a, b,** Exemplary charge stability diagrams taken at the Q6t-Q7 (3,1)-(2,2) charge interdot line. The two maps are taken at the diagonally opposite points of the two-dimensional barrier scan, at t_{6t7}, j_{6t7} = (-330, -510) and (-270, -380) mV, respectively. Orange lines represent a fit to the map following the procedure shown in ref. [S25]. A black circle identifies the fitted centre of the interdot, and the arrow illustrates the size in voltage of the interdot line. **c, d,** Size of the Q6b-Q7 (3,1)-(2,2) charge interdot line as a function of the two virtual barriers. **e, f,** Same for the Q6t-Q7 (3,1)-(2,2) charge interdot line. Outliers in the plot are due to non-accurate fits of the image.

Suppl. Note 11. TWO-AXIS CONTROL OF THE Q6B-Q5M INTERACTION

We repeat the tunnel coupling experiments considering the double-dot pair Q6b and Q5m by defining the virtual barriers t_{6b5} and j_{6b5} starting from the relative UB4 and LB6 barriers (Suppl. Fig. **11**):

$$\begin{pmatrix} \mathrm{P5} \\ \mathrm{P6} \\ \mathrm{UB4} \\ \mathrm{LB6} \\ \mathrm{SE\_P} \end{pmatrix} = \begin{pmatrix} -1.63 & -0.58 \\ -1.61 & -0.48 \\ 1 & 0 \\ 0 & 1 \\ -0.43 & -0.02 \end{pmatrix} \begin{pmatrix} \mathrm{t}_{6b5} \\ \mathrm{j}_{6b5} \end{pmatrix}$$

We observe that the limited sensitivity at the interdot hinders a quantitative tunnel coupling analysis. However, at a qualitative level, we still observe the expected pattern with the size of the interdot tunable by both barriers.

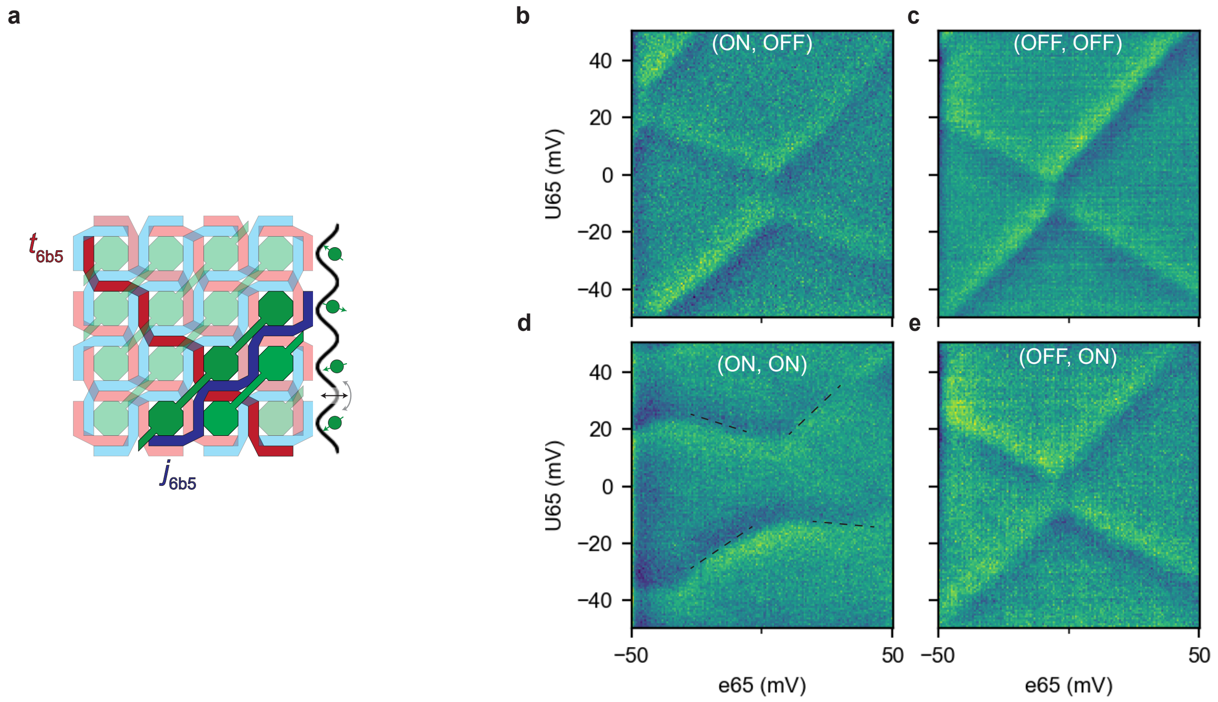

Supplementary Figure 11. **Two-axis control of the Q6b-Q5m interdot coupling.** **a** Schematic of the crossbar indicating the two intersecting virtual barriers (in red and blue) controlling the Q6b-Q5m interaction. **b-e** Charge stability diagrams at different barrier voltages: **(b)** (t_{6b5}, j_{6b5}) = (-330, -380) mV, **(c)** (-270, -380) mV, **(d)** (-270, -510) mV, **(e)** (-330, -510) mV. In **(d)**, in the high interdot coupling regime, we add dashed lines as guide for the eyes on weakly visible transition lines. Here, we display the signal from the SE charge sensor after subtraction of a background.

Suppl. Note 12. ELECTRON TEMPERATURE EXTRACTION

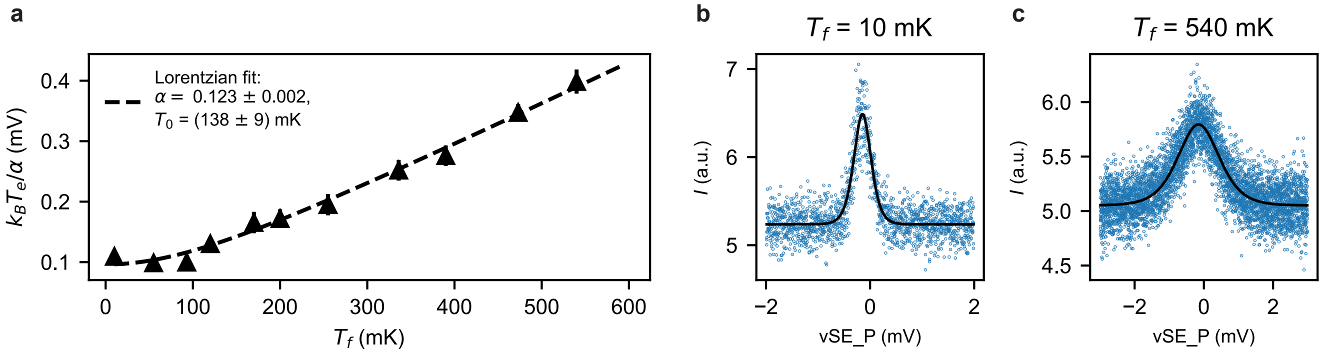

Supplementary Figure 12. Electron temperature extraction. a Coulomb peak width as a function of fridge temperature. **b, c.** Exemplary data (scatter points) and best fits (black lines) collected at $T_f = 10$ mK and $T_f = 540$ mK, respectively. For every temperature, we perform five plunger sweeps across the Coulomb peak (each averaged 500 times) and, by fitting the curves, we obtain five different estimates for the Coulomb peak width. In **(a)**, we show the average width. The depicted standard deviation is determined by considering the standard deviation from the single fit itself or the standard deviation between the five different fitting estimates, depending on which is dominant.

We estimate the electron temperature of our setup by fitting a temperature-broadened Coulomb peak of the SE charge sensor. We choose a source-drain voltage V_{SD} and coupling energy to the leads $h\Gamma$ such that $h\Gamma, eV_{\mathrm{SD}} << k_B T_e$, with h and k_B the Plank and Boltzmann constants, respectively and Γ the lead-dot tunnel rate. The current I at the Coulomb peak is fitted with the Lorentzian distribution

$$I_{model} = a \cdot \cosh^{-2}\left(\frac{\alpha_{sensor} \cdot \epsilon}{2k_B T_e}\right) + c, \tag{5}$$

where a is an amplitude prefactor, α_{sensor} is the lever arm of the sensor plunger gate, ϵ is the plunger gate voltage and c an offset. The full width half maximum (FWHM) of the peak is therefore given by

$$\mathrm{FWHM} = \frac{k_B T_e}{\alpha_{sensor}} = \frac{k_B \sqrt{T_0^2 + T_f^2}}{\alpha_{sensor}}, \tag{6}$$

where T_f is the nominal fridge temperature and T_0 the base electron temperature [S26]. We use the FWHM dependence on the nominal fridge temperature to extract both T_0 and α_{sensor}. The result can be seen in Suppl. Fig. **12** with two exemplary fits for 10 and 540 mK. The fit with Equation 6 results in $T_0 = 138 \pm 9$ mK and $\alpha_{sensor} = 0.123 \pm 0.002$ eV/V, which is consistent with an independent lever arm extraction from Coulomb diamonds.

Suppl. Note 13. DETUNING LEVER ARM EXTRACTION

For an accurate estimation of the interdot tunnel coupling, we evaluate the quantum dot detuning lever arm by fitting the sensor signal S_{21} with a thermally limited polarisation line (Suppl. Fig. **13**) as described in [S27]:

$$S_{model} = S_0 \pm \delta S \frac{\epsilon}{\Omega} \cdot \tanh\left(\frac{\Omega}{2k_B T_e}\right) + \frac{\partial S}{\partial \epsilon}\epsilon \tag{7}$$

with S_0 the background signal of the charge sensor, δS the signal amplitude, ϵ the detuning energy, Ω the energy difference between the two levels and the term $\frac{\partial S}{\partial \epsilon}\epsilon$ a linear slope due to cross-talk to the charge sensor. In the low-tunnelling regime, we can approximate $\Omega = \sqrt{\epsilon^2 + 4t^2} \approx \epsilon$, which reduces Equation 7 to

$$S_{\text{model}} = S_0 \pm \delta S \cdot \tanh\left(\frac{\alpha_{QD} \cdot \epsilon}{2k_B T_e}\right) + \frac{\partial S}{\partial \epsilon}\epsilon. \tag{8}$$

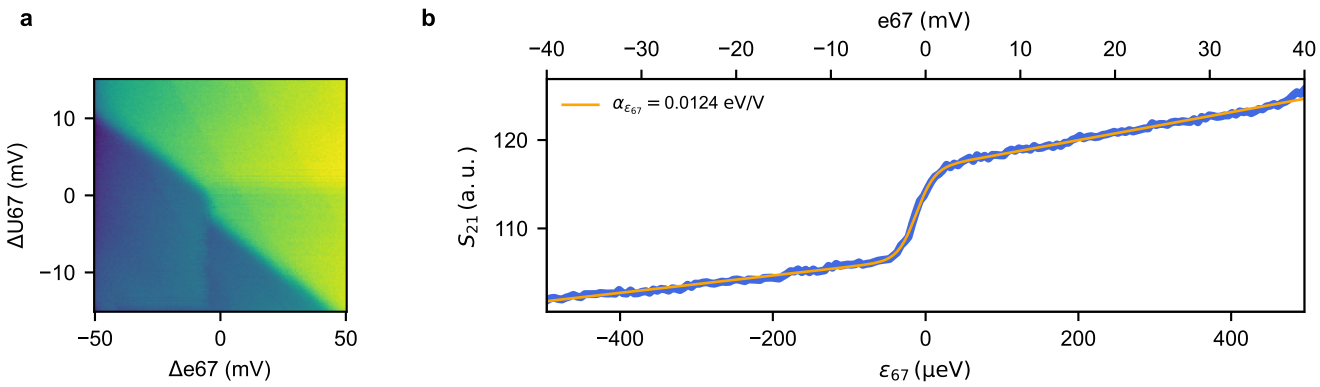

Supplementary Figure 13. **Temperature limited polarisation line. a,** Charge stability diagram of the uncoupled Q6b-Q7 at the (3,1)-(2,2) charge interdot. In this configuration, the tunnel time in Q6b is small with respect to the ramp time of the detuning axis, therefore the interdot is extended and the Q6b addition line is not clearly visible in the map. **b,** Thermally limited polarisation line (blue trace) taken at the centre of the interdot shown in **(a)**. From the best fit (orange line), we obtain the detuning lever arm $\alpha_{\epsilon_{67}} = 0.012(4)$ eV/V.

Suppl. Note 14. TUNE-UP OF THE CROSSBAR ARRAY IN THE FEW-HOLE REGIME

In Suppl. Fig. **14**, we display the charge stability diagrams obtained for the first tune-up of the device with the quantum dots in the few-holes regime. These maps are part of the data sets that are used for the analysis presented in Suppl. Fig. **5**.

Suppl. Note 15. GATE VOLTAGES OF THE CROSSBAR IN THE ODD-CHARGE REGIME

Suppl. Note 16. CHARACTERISATION OF THE VARIABILITY OF THE QUANTUM DOT ARRAY

We have characterised the level of homogeneity in the array considering two different metrics.
First, we consider the onset voltage of the first hole in each quantum dot, as displayed in Suppl. Figs. **16**a, b. We obtain an average first-hole voltage of -1660 $\pm$ 290 mV, that indicates a rather high variability across the quantum dot array. We also observe that a more negative voltage is required to accumulate the first hole when going toward the center of the array (e.g. under P4 and P5). This trend is anticorrelated with the voltages set to the LB barrier gates, that run in parallel to the plunger gates (Suppl. Fig, **16**c). The higher values of LB4 and LB5 lead to more negative values of the plunger gates within the array, due to cross capacitance. We note that the need to set LB4 and LB5 to a more positive voltage comes from the demand to maintain the charge sensors well separated by the array. In practice, we have observed that the needs to preserve the quality of the charge sensors and to set the interdot tunnel barriers seem conflicting. Therefore, we suggest in future designs to dedicate to one gate the function to isolate the sensor to the dots and to another gate to determine the interdot barriers.
A second metric to asses the variability in size and in dot lever arm relies on evaluating the homogeneity in quantum dot addition voltages, i.e. the spacing between two consecutive charge transition lines [S28]. We extract the charge addition voltages of all the quantum dots from the corresponding stability diagrams (Suppl. Fig. **17**), and extract the averaged spacing of the first and second hole to be 51 ± 6 mV and 50 ± 9, respectively, indicating a $\sim 10 - 20\%$ variability of the quantum dot confinement properties across the array.
Overall, this demonstrates that, while quantum dots are accumulated at rather different voltages under each plunger gate, their size and plunger gate coupling are similar, owing to the grid structure of our design.

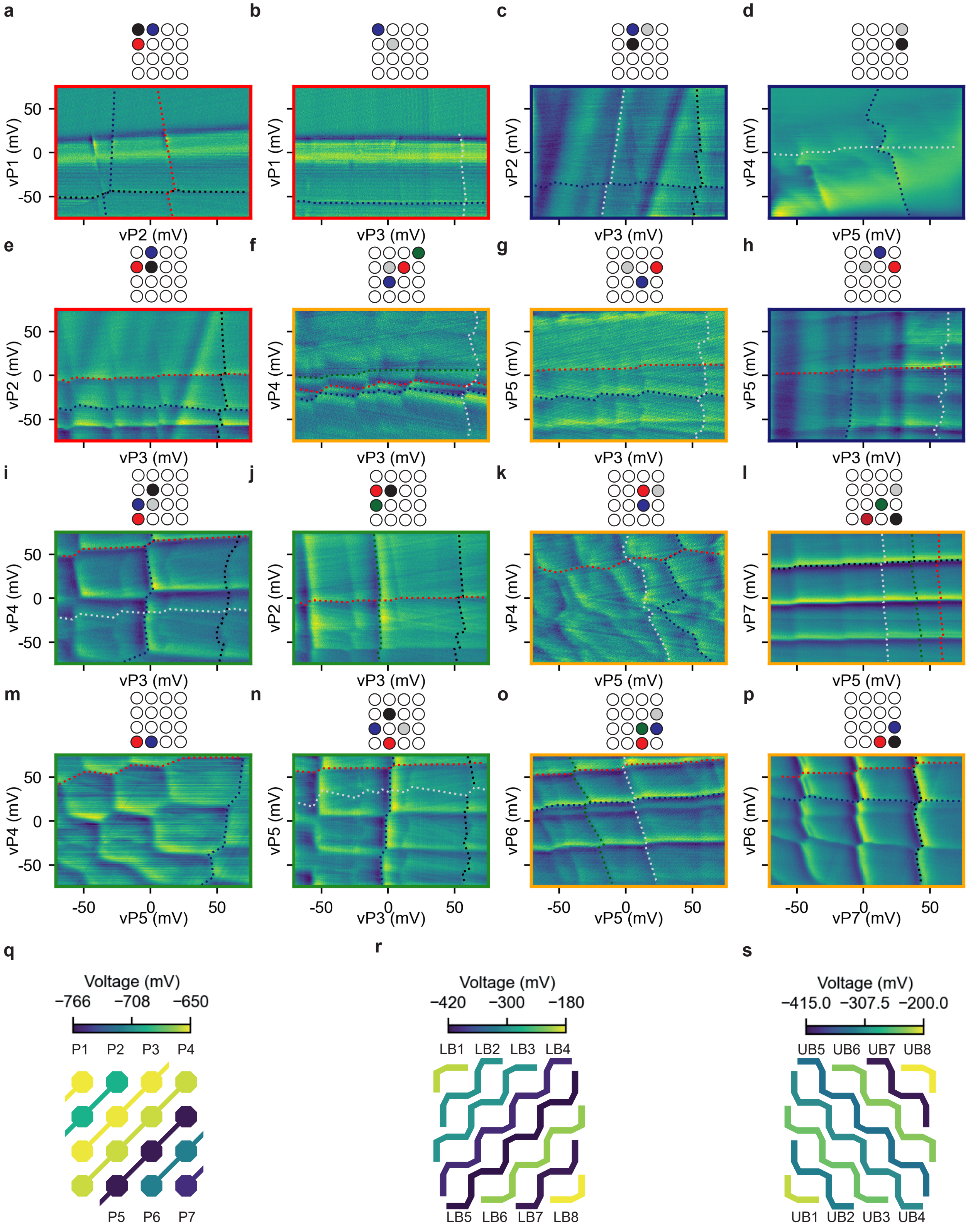

Supplementary Figure 14. Tune-up of the crossbar array in the few-holes regime. a-p, Charge stability diagrams (raw sensor signal after subtraction of a background) showcasing the 16 quantum dot system in the few-hole regime. These measurements are taken in the first tune-up of the crossbar array. In each map, the first visible transition lines from the right or the top are labelled and assigned to the relative quantum dot by dashed lines with colours defined in the schematic at the top. The identification is performed via the results of Suppl. Fig. **5**. The colour of the panel frame identifies the sensor used: NW in red, NE in blue, SW in green, and SE in ochre. **q-s,** Schematics of the voltage applied at all crossbar gates at this phase of the experiment. The voltages are here optimised to circumvent stray dots.

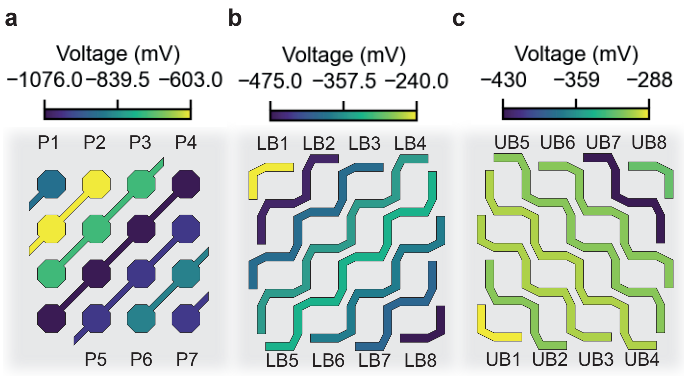

Supplementary Figure 15. **Crossbar gate voltages. a-c,** Voltages applied at the real P, LB and UB gates, respectively, when the system is tuned in the odd charge occupation regime.

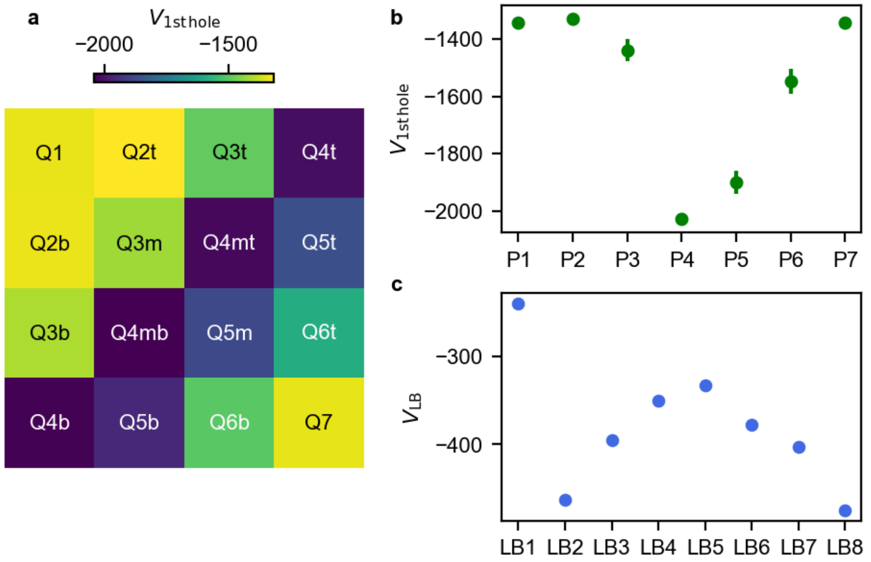

Supplementary Figure 16. **Onset voltages for the first hole. a,** Colormap of the virtual plunger voltages required to accumulate the first hole in each quantum dot. **b,** Averages first hole virtual plunger voltages. The error bars represent the standard deviations around the mean. We note that, while the variability of dot voltages for the same plunger is rather low, the overall variability of 290 mV remains high. **c,** Voltages of the LB gates in the odd charge regime.

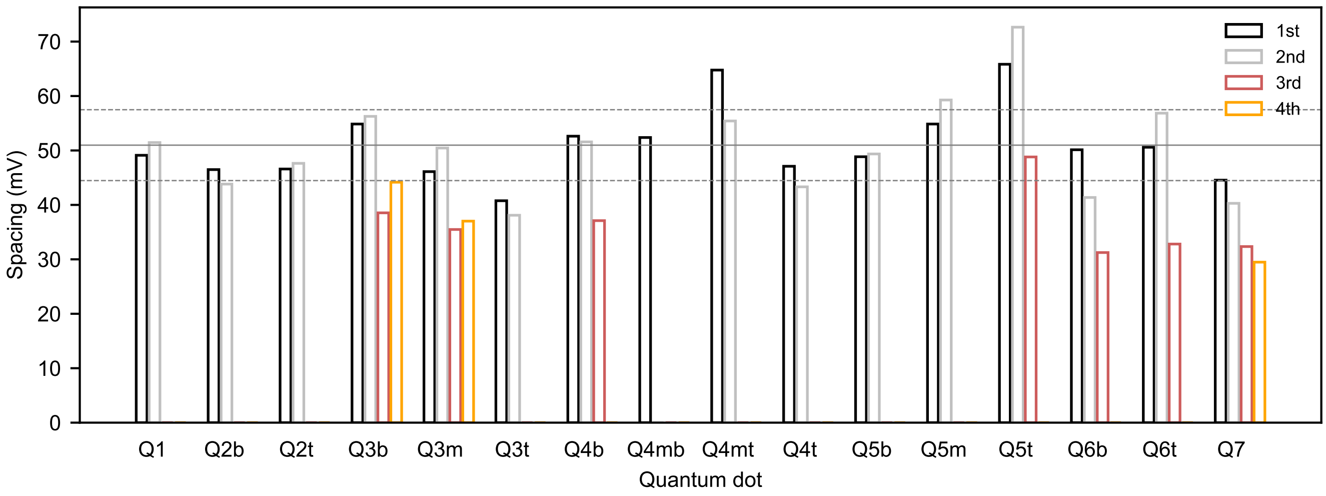

Supplementary Figure 17. **Charge Addition voltages of all the quantum dots.** Histogram of the addition voltages of each quantum dot considering the first spacings, when detectable in the already existing datasets. The three horizontal lines identify the spread of the first hole addition voltage of 51 ± 6 mV.

[S1] R. Van Meter and D. Horsman, A blueprint for building a quantum computer, Commun. ACM **56**, 84–93 (2013).

[S2] D. P. Franke, J. S. Clarke, L. M. Vandersypen, and M. Veldhorst, Rent's rule and extensibility in quantum computing, Microprocess. Microsyst. **67**, 1 (2019).

[S3] F. Arute, K. Arya, R. Babbush, D. Bacon, J. C. Bardin, R. Barends, R. Biswas, S. Boixo, F. G. S. L. Brandao, D. A. Buell, B. Burkett, Y. Chen, Z. Chen, B. Chiaro, R. Collins, W. Courtney, A. Dunsworth, E. Farhi, B. Foxen, A. Fowler, C. Gidney, M. Giustina, R. Graff, K. Guerin, S. Habegger, M. P. Harrigan, M. J. Hartmann, A. Ho, M. Hoffmann, T. Huang, T. S. Humble, S. V. Isakov, E. Jeffrey, Z. Jiang, D. Kafri, K. Kechedzhi, J. Kelly, P. V. Klimov, S. Knysh, A. Korotkov, F. Kostritsa, D. Landhuis, M. Lindmark, E. Lucero, D. Lyakh, S. Mandrà, J. R. McClean, M. McEwen, A. Megrant, X. Mi, K. Michielsen, M. Mohseni, J. Mutus, O. Naaman, M. Neeley, C. Neill, M. Y. Niu, E. Ostby, A. Petukhov, J. C. Platt, C. Quintana, E. G. Rieffel, P. Roushan, N. C. Rubin, D. Sank, K. J. Satzinger, V. Smelyanskiy, K. J. Sung, M. D. Trevithick, A. Vainsencher, B. Villalonga, T. White, Z. J. Yao, P. Yeh, A. Zalcman, H. Neven, and J. M. Martinis, Quantum supremacy using a programmable superconducting processor, Nature **574**, 505 (2019).

[S4] S. G. J. Philips, M. T. Madzik, S. V. Amitonov, S. L. de Snoo, M. Russ, N. Kalhor, C. Volk, W. I. L. Lawrie, D. Brousse, L. Tryputen, B. P. Wuetz, A. Sammak, M. Veldhorst, G. Scappucci, and L. M. K. Vandersypen, Universal control of a six-qubit quantum processor in silicon, Nature **609**, 919 (2022).

[S5] M. Y. Lanzerotti, G. Fiorenza, and R. A. Rand, Microminiature packaging and integrated circuitry: The work of e. f. rent, with an application to on-chip interconnection requirements, IBM J. Res. Dev. **49**, 777 (2005).

[S6] N. W. Hendrickx, W. I. Lawrie, M. Russ, F. van Riggelen, S. L. de Snoo, R. N. Schouten, A. Sammak, G. Scappucci, and M. Veldhorst, A four-qubit germanium quantum processor, Nature **591**, 580 (2021).

[S7] P.-A. Mortemousque, E. Chanrion, B. Jadot, H. Flentje, A. Ludwig, A. D. Wieck, M. Urdampilleta, C. Bäuerle, and T. Meunier, Coherent control of individual electron spins in a two-dimensional quantum dot array, Nat. Nanotechnol. **16**, 296 (2021).

[S8] L. M. K. Vandersypen, H. Bluhm, J. S. Clarke, A. S. Dzurak, R. Ishihara, A. Morello, D. J. Reilly, L. R. Schreiber, and M. Veldhorst, Interfacing spin qubits in quantum dots and donors—hot, dense, and coherent, npj Quantum Inf. **3** (2017).

[S9] J. A. Morton and W. J. Pietenpol, The technological impact of transistors, PIRE **46**, 955 (1958).

[S10] P. Christie and D. Stroobandt, The interpretation and application of rent's rule, IEEE Trans. Very Large Scale Integr. (VLSI) Syst. **8**, 639 (2000).

[S11] R. Li, L. Petit, D. P. Franke, J. P. Dehollain, J. Helsen, M. Steudtner, N. K. Thomas, Z. R. Yoscovits, K. J. Singh, S. Wehner, L. M. K. Vandersypen, J. S. Clarke, and M. Veldhorst, A crossbar network for silicon quantum dot qubits, Sci. Adv. **4**, eaar3960 (2018).

[S12] T. Hensgens, T. Fujita, L. Janssen, X. Li, C. J. Van Diepen, C. Reichl, W. Wegscheider, S. Das Sarma, and L. M. K. Vandersypen, Quantum simulation of a fermi–hubbard model using a semiconductor quantum dot array, Nature **548**, 70 (2017).

[S13] A. R. Mills, D. M. Zajac, M. J. Gullans, F. J. Schupp, T. M. Hazard, and J. R. Petta, Shuttling a single charge across a one-dimensional array of silicon quantum dots, Nat. Commun. **10** (2019).

[S14] W. Ha, S. D. Ha, M. D. Choi, Y. Tang, A. E. Schmitz, M. P. Levendorf, K. Lee, J. M. Chappell, T. S. Adams, D. R. Hulbert, E. Acuna, R. S. Noah, J. W. Matten, M. P. Jura, J. A. Wright, M. T. Rakher, and M. G. Borselli, A flexible design platform for Si/SiGe exchange-only qubits with low disorder, Nano Lett. **22**, 1443 (2022).

[S15] P. L. Bavdaz, H. G. J. Eenink, J. van Staveren, M. Lodari, C. G. Almudever, J. S. Clarke, F. Sebasatiano, M. Veldhorst, and G. Scappucci, A quantum dot crossbar with sublinear scaling of interconnects at cryogenic temperature, npj Quantum Information **8**, 86 (2022).

[S16] M. Meyer, C. Déprez, T. R. van Abswoude, I. N. Meijer, D. Liu, C.-A. Wang, S. Karwal, S. Oosterhout, F. Borsoi, A. Sammak, N. W. Hendrickx, G. Scappucci, and M. Veldhorst, Electrical control of uniformity in quantum dot devices, Nano Letters 10.1021/acs.nanolett.2c04446 (2023).

[S17] C. H. Yang, A. Rossi, N. S. Lai, R. Leon, W. H. Lim, and A. S. Dzurak, Charge state hysteresis in semiconductor quantum dots, Applied Physics Letters **105**, 183505 (2014), https://doi.org/10.1063/1.4901218.

[S18] P. Harvey-Collard, B. D'Anjou, M. Rudolph, N. T. Jacobson, J. Dominguez, G. A. Ten Eyck, J. R. Wendt, T. Pluym, M. P. Lilly, W. A. Coish, M. Pioro-Ladrière, and M. S. Carroll, High-fidelity single-shot readout for a spin qubit via an enhanced latching mechanism, Phys. Rev. X **8**, 021046 (2018).

[S19] H. G. J. Eenink, L. Petit, W. I. L. Lawrie, J. S. Clarke, L. M. K. Vandersypen, and M. Veldhorst, Tunable Coupling and Isolation of Single Electrons in Silicon Metal-Oxide-Semiconductor Quantum Dots, Nano Lett. **19**, 8653 (2019).

[S20] W. I. L. Lawrie, H. G. J. Eenink, N. W. Hendrickx, J. M. Boter, L. Petit, S. V. Amitonov, M. Lodari, B. Paquelet Wuetz, C. Volk, S. G. J. Philips, G. Droulers, N. Kalhor, F. van Riggelen, D. Brousse, A. Sammak, L. M. K. Vandersypen, G. Scappucci, and M. Veldhorst, Quantum dot arrays in silicon and germanium, Appl. Phys. Lett. **116**, 080501 (2020).

[S21] X. Xue, M. Russ, N. Samkharadze, B. Undseth, A. Sammak, G. Scappucci, and L. M. K. Vandersypen, Quantum logic with spin qubits crossing the surface code threshold, Nature **601**, 343 (2022).

[S22] N. Hendrickx, D. Franke, A. Sammak, G. Scappucci, and M. Veldhorst, Fast two-qubit logic with holes in germanium, Nature **577**, 487 (2020).

[S23] M. Russ, D. M. Zajac, A. J. Sigillito, F. Borjans, J. M. Taylor, J. R. Petta, and G. Burkard, High-fidelity quantum gates in Si/SiGe double quantum dots, Phys. Rev. B **97**, 085421 (2018).

[S24] D. Loss and D. P. DiVincenzo, Quantum computation with quantum dots, Phys. Rev. A **57**, 120 (1998).

[S25] C. J. van Diepen, P. T. Eendebak, B. T. Buijtendorp, U. Mukhopadhyay, T. Fujita, C. Reichl, W. Wegscheider, and L. M. K. Vandersypen, Automated tuning of inter-dot tunnel coupling in double quantum dots, Appl. Phys. Lett. **113**, 033101 (2018).

[S26] L. Petit, J. Boter, H. Eenink, G. Droulers, M. Tagliaferri, R. Li, D. Franke, K. Singh, J. Clarke, R. Schouten, V. Dobrovitski, L. Vandersypen, and M. Veldhorst, Spin Lifetime and Charge Noise in Hot Silicon Quantum Dot Qubits, Phys. Rev. Lett. **121**, 076801 (2018).

[S27] L. DiCarlo, H. J. Lynch, A. C. Johnson, L. I. Childress, K. Crockett, C. M. Marcus, M. P. Hanson, and A. C. Gossard, Differential Charge Sensing and Charge Delocalization in a Tunable Double Quantum Dot, Phys. Rev. Lett. **92**, 226801 (2004).

[S28] D. M. Zajac, T. M. Hazard, X. Mi, E. Nielsen, and J. R. Petta, Scalable gate architecture for a one-dimensional array of semiconductor spin qubits, Phys. Rev. Appl. **6**, 054013 (2016).
